# Physics-Guided Machine Learning for Predicting Critical Salt Concentration and pH in Sandstone Reservoirs

**DOI:** 10.1021/acsomega.6c06045

**Published:** 2026-08-20

**Authors:** Sami Abderraouf Belkhir, Rizwan Muneer, Mariam Shakeel, Younes Alblooshi, Muhammad Rehan Hashmet

**Affiliations:** † Department of Chemical and Petroleum Engineering, 11239 United Arab Emirates University, 15551 Al Ain, United Arab Emirates; ‡ College of Engineering and Science, 534894 Hamad Bin Khalifa University, Education City, Doha 34110, Qatar; § Department of Civil and Environmental Engineering, College of Engineering, 61780 Qatar University, Doha 34110, Qatar

## Abstract

Fines migration is a critical formation-damage mechanism in sandstone reservoirs, causing permeability reduction and injectivity loss during water flooding and injection operations. The onset of fines mobilization is governed by various key physicochemical thresholds, including critical salt concentration (CSC) and critical pH. These parameters control the balance between attractive and repulsive surface forces at the fine–brine–rock interface through electrical double-layer expansion. Although Derjaguin–Landau–Verwey–Overbeek (DLVO)-based modeling provides mechanistic insight, high-throughput application requires repeated calculations and extensive system-specific electrokinetic characterization. This study presents a physics-informed machine-learning framework that provides rapid and accurate predictions of both CSC and critical pH directly from reservoir, brine, and mineralogical descriptors. The main contribution is the development and systematic evaluation of machine-learning surrogate models capable of reproducing DLVO-derived fines-mobilization thresholds without repeated electrokinetic calculations. Nine ensemble algorithms were evaluated and optimized via Bayesian hyperparameter tuning using Optuna. Model performance was assessed using *R* ^2^, RMSE, and MAE, with multicollinearity addressed through Pearson correlation analysis prior to training. The Extra Trees regressor demonstrated superior performance for both targets, achieving *R* ^2^ = 0.9999, RMSE = 0.99, and MAE = 0.42 for CSC prediction, and *R* ^2^ = 0.9999, RMSE = 0.12, and MAE = 0.03 for critical pH on independent, unseen test sets. Random Forest and CatBoost models were also able to achieve acceptable match between the true and predicted values of total potential; however, AdaBoost and XGBRF failed to capture the nonlinear behavior of electrostatic transitions. The optimized Extra Trees surrogate reproduced the DLVO-derived total interaction potential, *V*_t_, with the highest accuracy among the tested models. CSC and critical pH are therefore obtained through a postprocessing threshold-extraction step applied to the predicted *V*_t_ profiles after screening, rather than as independent scalar outputs detached from the DLVO energy criterion. The proposed framework therefore combines mechanistic consistency with computational efficiency and enables rapid screening of fines-migration risks within the investigated ionic-strength, pH, zeta potential, and separation-distance ranges. It can support the selection of injection conditions that minimize permeability impairment and injectivity loss while reducing dependence on laboratory-intensive electrokinetic characterization.

## Graphical abstract

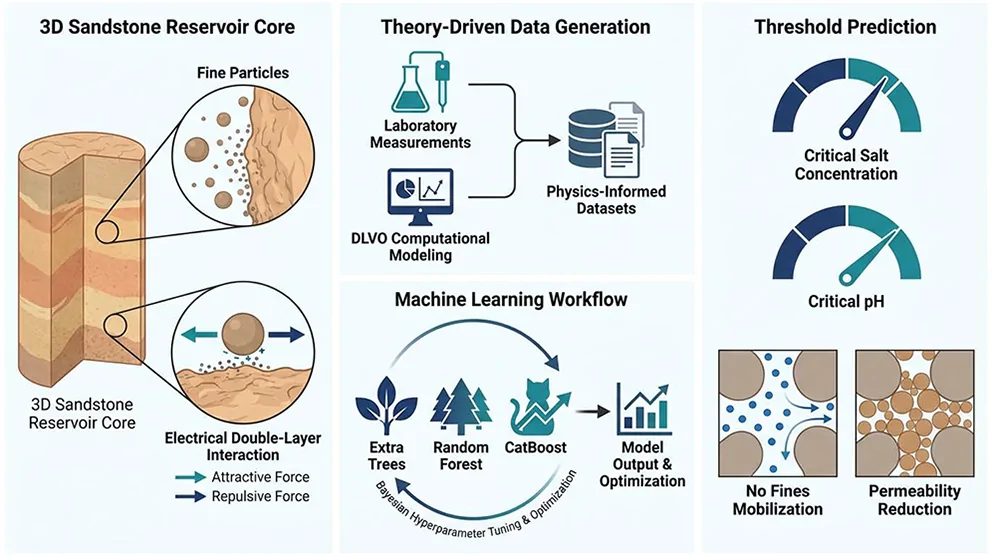

## 1 Introduction

Fines migration in sandstones is a phenomenon of detachment, mobilization (straining), and deformation of ultrafine quartz and clay particles (e.g., kaolinite, illite, chlorite) residing on grain surfaces or in pore spaces.^1–3^ They can be removed and transported by filtration and fluid flow, which results in the blockage of pore throats, reduction in the effective pore space, an increase in wellbore pressure, and therefore decline in injectivity and productivity.^4^ The damage caused by fines is governed by various “critical” operating conditions, such as the critical salt concentration (CSC), critical pH, and critical flow rate, because they affect the balance between the attractive and repulsive surface forces at the fine–brine–rock interface.^5–7^ The electrical double layer (EDL) around grains and fines expands (repulsive force) at a low salinity (below the CSC) or at a critical pH (more alkaline) and facilitates the detachment and migration even at low flow rates, whereas at higher salinity and/or neutral pH, the ED is compressed and attractive forces dominate.^8–13^ These processes and their impact including permeability loss, pressure drop, and potential for formation damage during water injection, well completion, and acidizing are well-understood and can be quantified using surface-force calculations.^14–16^

There are numerous operational scenarios where fines migration occurs, including aquifer recharging by external water sources,^17–19^ underground disposal of formation water, groundwater flow, injection of drilling and completion fluids, changes in injection-water salinity,^20^ alkaline flooding,^21^ and high-rate production or injection.^22^ The physics behind each process is the same, where fines are affected by surface forces, and attraction of fines to grain surfaces is enhanced or reduced based on changes in the EDL thickness and grain surface potentials.^23^ Upon decreasing the salinity of the injected brine below the CSC or increasing the pH above the critical pH, repulsive EDL force can dominate attractive and gravitational forces, resulting in fines release, mobilization, and straining in constricted pore throats.^24,25^ The consequent permeability loss and pressure drop can be significant if the fines concentration is large or if pore-throat geometry favors bridging.^26,27^

To predict these force balances, DLVO modeling describes the total energy between fines and sand grains as the sum of London–van der Waals attraction, EDL repulsion, and short-range Born repulsion.^28–31^ The model requires specific parameters such as measured zeta potentials of fines–sand–brine system, brine composition/ionic strength, and fines average size, given that they control the magnitude and sign of the energy over nanometric distances between fines and sand. In sandstone systems, the total potential energy can be calculated (with measured zeta potentials and reasonable Hamaker constants); the transition from net attraction (negative) to net repulsion (positive) describes the critical conditions for fines release.^32^ This approach has been used to predict CSC for monovalent and divalent brines and critical pH for alkaline conditions (in sandstone cores), with a close agreement between model predictions and core flood data and zeta potential measurements. DLVO modeling can serve as an accurate predictive tool but requires extensive experimentation to define the fine size and zeta potential of sand–fine–brine systems as a function of salinity and pH, which is time- and resource-intensive. Moreover, repeated DLVO calculations and electrokinetic measurements become impractical when many combinations of brine composition, mineralogy, particle size, temperature, and pH must be screened. A computationally efficient surrogate is therefore needed to retain the physical consistency of DLVO modeling while enabling rapid evaluation of fines-mobilization thresholds over a broad range of reservoir conditions.

Machine learning (ML) is increasingly becoming a mainstream method for prediction, optimization, and risk-based decision-making in petroleum and reservoir engineering. Recent reviews present applications in production prediction, fracking, water/chemical/gas injection, and reservoir monitoring with key advantages, including rapid surrogate model testing, identification of nonlinear features, and uncertainty quantification, compared to physics-based models.^33,34^ At the same time, surrogate models are increasingly used to accelerate multiscenario reservoir management and optimization instead of costly high-accuracy models, while new types of deep learning are used in pore-to-field-scale modeling, and even molecular-scale predictions for interfacial properties.^35^ In general, these works suggest that reliable ML models, combined with physically meaningful features, can be used to effectively screen and predict across a broad spectrum of reservoir conditions.^36^

Within the formation-damage domain, ML is beginning to address fine-induced damage and pore-clogging effects. A recent study has used pattern recognition and ML techniques to model damage patterns in sandstone after smart-water flooding, where brine properties and process conditions are correlated with damage patterns.^37^ At the pore scale, deep neural networks have been applied to predict pore clogging and changes in porosity from an image-based representation of a granular medium to enable rapid prediction of fines clogging.^38^ More broadly, ML prediction models have also identified and sorted clogging regimes in porous media using dimensionless parameters and predicted the likelihood of fine-particle retention to estimate permeability declines, thus enhancing the risk assessment of formation-damage operations.^39^ Related ML research in formation-damage prediction (e.g., scale deposition and permeability) also supports the potential to learn the nonlinear characteristics of water chemistry, operating conditions, and formation-damage metrics when combined with suitable features and validations.^40^

Existing machine-learning applications in formation damage and reservoir engineering have primarily focused on permeability prediction, waterflood-efficiency screening, pore-clogging classification, production response, and general formation-damage risk assessment. These studies demonstrate the value of data-driven models for learning nonlinear relationships among fluid chemistry, porous-media structure, and reservoir performance. However, previous ML studies have not explicitly coupled DLVO-derived interaction potential surfaces with a unified threshold-extraction workflow for both critical salt concentration (CSC) and critical pH. This distinction is important because CSC and critical pH correspond to different operational controls on fines mobilization: CSC is mainly governed by ionic-strength-controlled electrical double-layer compression/expansion, whereas critical pH is governed by surface-charge modification through protonation and deprotonation reactions at mineral surfaces. More importantly, earlier studies have generally focused on the consequences of fines migration, such as particle retention, pore clogging, and permeability reduction, whereas direct ML prediction of the physicochemical thresholds initiating fines release remains insufficiently addressed.

The present study addresses this gap by developing a physics-informed ML surrogate trained on DLVO-generated interaction potential data and by explicitly converting the predicted total interaction potential, *V*_t_, into actionable CSC and critical pH thresholds through zero-crossing detection. The specific contributions include (i) generating physically consistent labeled data sets from mechanistic DLVO calculations; (ii) simultaneously predicting two principal fines-mobilization thresholds rather than only the resulting formation damage; (iii) systematically comparing nine ensemble-learning algorithms; and (iv) optimizing the models through Bayesian hyperparameter tuning and evaluating them using independent, unseen test data sets. The curated physics-labeled data sets ensure physicochemical consistency and enable rapid threshold prediction within the validated training domain.

pH and ionic strength are attractive descriptors for field deployment because they are routinely available from injected-water and produced-water analyses. Zeta potential, in contrast, provides a direct electrokinetic description of the mineral–brine interface but requires specialized measurements, such as electrophoretic light scattering or streaming-potential experiments, and is strongly system-specific. Therefore, excluding zeta potential, where supported by correlation analysis, can improve the practical usability of the model. The resulting framework is intended as a preliminary DLVO-based screening tool rather than a complete replacement for detailed DLVO analysis. Additional DLVO calculations and experimental characterization remain necessary for new mineralogies, temperatures, brine compositions, or other conditions outside the investigated ionic strength, pH, zeta potential, and separation-distance ranges.

## 2 Methodology

### 2.1 Data Acquisition

The data sets used in this study are physics-derived DLVO simulation data sets, that were validated through experimental measurements. For each prescribed physicochemical condition, the total interaction potential, V_t_, was calculated from the sum of van der Waals attraction, electrostatic double-layer repulsion, and short-range Born repulsion over the corresponding separation-distance grid. The final number of observations therefore reflects the sampling design used in the DLVO parameter sweep. Salinity-dependent data for critical salt concentration (CSC) prediction were obtained from the DLVO models reported by Muneer et al.^8,32^ Here, salinity values between 0.04 and 0.2 M were considered and the corresponding zeta potential values were used in the DLVO equations to calculate the total interaction potential (V_t_). The zeta potential values used in the DLVO calculations were measured for dispersed sandstone particles in the corresponding brines and were used as an effective surface-potential representation of the fine–sand interaction. The resulting salinity data set contained 2850 data points generated by the model with ionic-strength values of 0.04–0.2 M, zeta potentials of −29.5 to −35.5, and separation distances of 0.25–10 nm.

Similarly, the pH-dependent data set was generated from the DLVO models presented by Muneer et al.^11,41^ For these calculations, pH conditions from 3 to 9 were used together with the reported zeta potential values to calculate *V*_t_. The processed data set contained 4163 model-generated data points covering pH values of 3–9, ζ-potential from −7.0 to −25.5 mV, and separation distances from 0.05 to 0.75 nm. Collectively, these DLVO-derived data sets provided the structured inputs required to train the ML models for predicting critical CSC and critical pH for fines-migration initiation.

In this ML framework, *h* is retained as an input feature because the model reconstructs *V*_t_(*h*) profiles. It is not an operational field control variable like pH, salinity, or ionic strength. For field screening, the model scans the prescribed h-grid and applies the selected stability criterion to the predicted *V*_t_ profiles.

The ranges for the investigated parameter were not selected arbitrarily. They were adopted from the experimentally validated electrokinetic and DLVO data sets reported in our previous studies^8,11,32,41^ and were selected to encompass the transition from net attractive to net repulsive interaction conditions associated with fines release. The ML models predict the total interaction potential as a function of ionic strength or pH and separation distance, from which the CSC and critical pH are identified at the attraction-to-repulsion transition. Conditions outside the investigated ranges were not included because they would require extrapolation of the experimentally established ζ-potential relationships beyond their validated limits. Other DLVO parameters, including the particle size, Hamaker constant, temperature, dielectric properties, and Born-repulsion parameters, were maintained at the values used in the validated source models and were therefore not treated as independent ML predictors. The complete ranges of the varied parameters and the values of the fixed physicochemical constants used to generate both DLVO-based data sets are summarized in Table S1. Practical examples of predicting the CSC and critical pH using experimental data are illustrated in Figure S1­(a,b), respectively.

The machine-learning workflow developed in this study follows a systematic, stepwise framework illustrated in Figure 1, beginning with the collection and processing of fines-migration data and culminating in the development of trained predictive models. These models were designed to estimate key response variables, including critical salt concentration and critical pH, and ultimately the onset of fines migrations, using fluid composition. A number of machine-learning algorithms were compared in order to identify the best predictive models for predicting the fines-migration behavior. Each model is useful for capturing various facets of relationships controlling this behavior. In this study, “physics-informed” refers to physics-supervised learning from DLVO-generated labels and does not denote a conventional physics-informed neural network in which governing equations are incorporated into the loss function. The ML surrogate was evaluated using independent, unseen DLVO-generated test data, while its experimental grounding was inherited from the source DLVO models previously validated against core flood observations and measured zeta potential data.^8,11,32,41^

**Figure 1:**
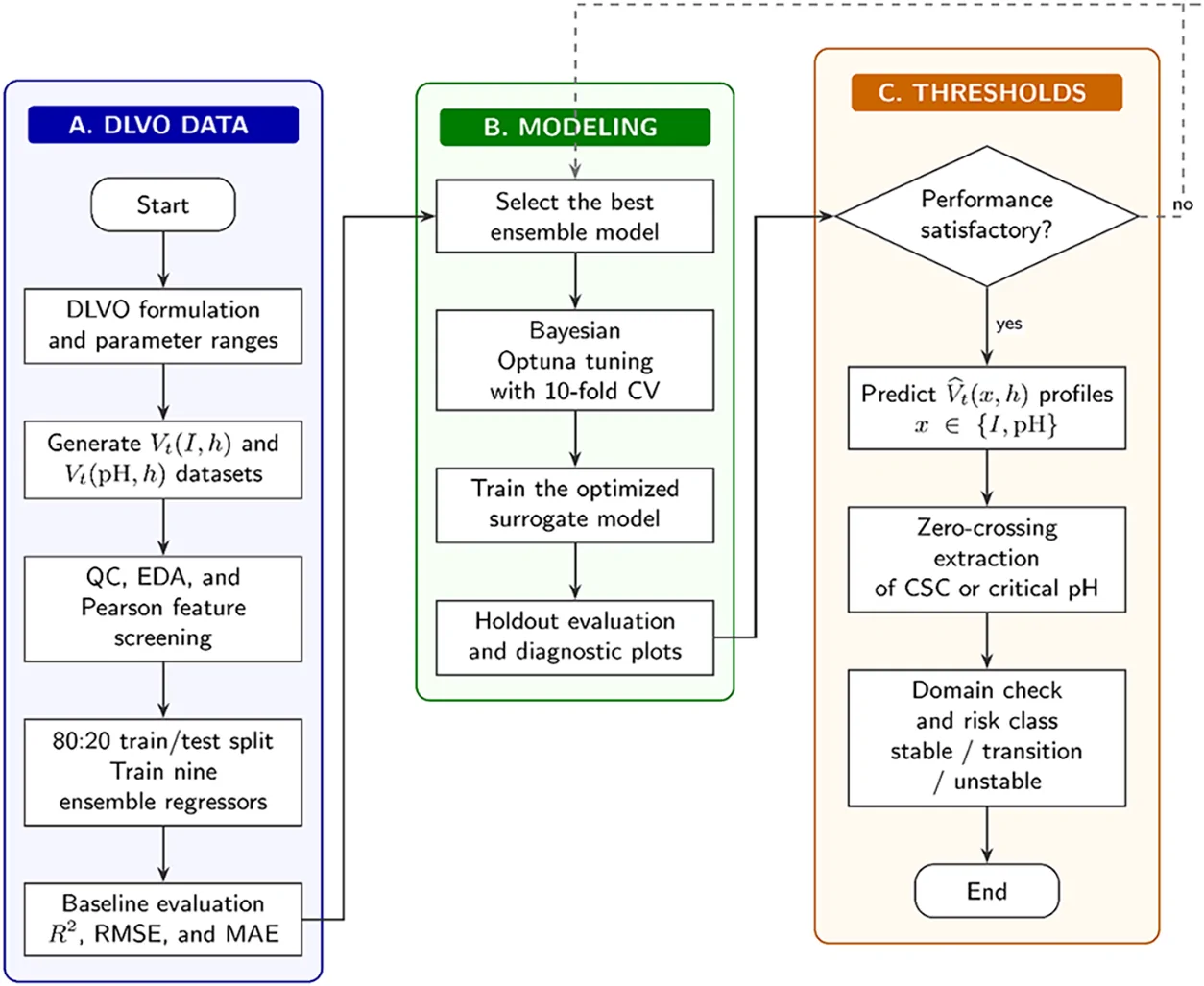
Workflow summarizing the methodology used in this study.

### 2.2 Data Preprocessing

Prior to model development, the data set was subjected to a structured preprocessing workflow implemented in Python using standard scientific libraries (e.g., Scikit-learn, Pandas, NumP). This workflow began with data import and inspection to verify completeness and internal consistency, followed by Exploratory Data Analysis (EDA) to examine the statistical distributions of input features and the target variables (critical salt concentration and critical pH), identify physical trends, and assess data balance across the parameter space. Pairwise scatter plots and marginal distributions were generated to investigate relationships among the variables and to verify the physical plausibility of the data set’s structure in the context of known DLVO behavior. Then, the Pearson correlation heatmaps were generated for the salt concentration and the pH data sets, respectively, in order to quantify dependencies between zeta potential, ionic strength or pH, separation distance, and total interaction potential with the explicit purpose of detecting and resolving multicollinearity prior to model training. To maximize model stability and interpretability, the final feature set was constructed by removing features that were nearly linearly dependent on retained predictors. For each data set, the initial candidate feature set included the controlling physicochemical variable, either pH or ionic strength, together with zeta potential and separation distance. Pearson correlation analysis was used to identify multicollinearity among the candidate predictors. Zeta potential was excluded because it was strongly correlated with pH and ionic strength, whereas the separation distance was retained because it independently controls the DLVO interaction potential. The final input sets therefore consisted of pH and separation distance for the pH data set and ionic strength and separation distance for the salinity data set. Because the data sets were generated from deterministic DLVO calculations, quality-control screening was limited to duplicate rows, missing values, nonfinite numerical outputs, unit-conversion inconsistencies, and negative separation distances. Then, all the numerical features were scaled to have zero mean and unit variance so that high-magnitude variables would not dominate the learning process.^42^ The data set was split in the ratio of 80:20 into training and test set, and 10-fold cross-validation using simple random partitioning was applied during hyperparameter tuning to check the generalization and reduce the risk of overfitting. The next section presents the results of EDA, correlation analysis, and model performance evaluation.

### 2.3 Ensemble Models

Advanced collection of ensemble-learning techniques was investigated and compared for prediction of fines migration under diverse scenarios. These approaches are highly suited for tabular regression problems since they can capture complicated nonlinear dependencies and higher-order interactions between input characteristics and provide good prediction resilience. Ensemble learning is a method of combining many models to enhance generalization and minimize variance, especially on complicated data.^43^ The ensemble models evaluated in this study included AdaBoost, random forest, gradient boosting, Extra Trees, bagging, CatBoost, LightGBM, XGBoost, and XGBRF. Collectively, these algorithms represent complementary tree-based learning strategies, including boosting, bagging, and randomized forest construction, thereby providing a comprehensive framework for assessing model performance across a wide range of fines migration scenarios. Their selection was motivated by their established effectiveness in structured data modeling and their ability to accommodate nonlinear physicochemical dependencies among reservoir, fluid, and operating parameters. The models used along with their brief description are outlined in Table 1.

**Table 1:** Machine-Learning Models Considered in the Predictive Framework

model	type	key feature	typical use	reference
AdaBoost	boosting	sequential weak learners to reduce error	improves prediction accuracy in structured data	^44^
Random Forest	bagging	ensemble of trees with random sampling	nonlinear regression and robust prediction	^45^
Gradient Boosting	boosting	sequential learning minimizing residuals	captures complex nonlinear relationships	^46,47^
Extra Trees	bagging	randomized splits to reduce variance	high-dimensional nonlinear problems	^48^
Bagging	bagging	averaging multiple models	improves model stability	^49^
CatBoost	boosting	handles categorical and nonlinear data	strong generalization with minimal tuning	^50^
LightGBM	boosting	leaf-wise tree growth, fast training	large-scale regression problems	^51^
XGBoost	boosting	regularized gradient boosting	high-accuracy predictive modeling	^52^
XGBRF	hybrid	combines RF and boosting strategies	balances variance and performance	^53^

### 2.4 Evaluation Metrics

To rigorously evaluate the predictive capability of the ensemble models, a combination of quantitative performance metrics and graphical diagnostics was employed for both training and independent test data sets. Three standard regression metrics were adopted: the coefficient of determination (*R* ^2^), root mean squared error (RMSE), and mean absolute error (MAE), defined respectively as

1 $$R^{2}=1-\frac{\sum_{i=1}^{n}{\left(y_{i}-{ŷ}_{i}\right)}^{2}}{\sum_{i=1}^{n}{\left(y_{i}-y\right)}^{2}}$$

2 $$\mathrm{RMSE}=\sqrt{\frac{1}{n}\sum_{i=1}^{n}{\left(y_{i}-{ŷ}_{i}\right)}^{2}}$$

3 $$\mathrm{MAE}=\frac{1}{n}\sum_{i=1}^{n}\left|y_{i}-{ŷ}_{i}\right|$$

where *y*_*i*_ is the DLVO-derived total interaction potential, *ŷ*_*i*_ is the model prediction, *y̅* is the mean of the target values, and *n* is the number of samples. *R* ^2^ measures the proportion of variance in the target explained by the model, with values approaching unity indicating near-perfect predictive fidelity; RMSE penalizes large deviations and is expressed in the same units as the target variable, making it sensitive to outliers; and MAE provides a linear, outlier-robust measure of average prediction error.^54^ In addition to these scalar metrics, graphical diagnostics including predicted-versus-actual parity plots and residual distribution analyses were employed. Scalar measures alone may not be enough to capture them, and were utilized to detect systematic bias, heteroscedasticity, and structural prediction errors, offering a more thorough evaluation of model reliability and generalization throughout the broad range of the study.^55^ The model performance was also tested with the use of learning curves and validation curves to identify underfitting or overfitting scenarios. These diagnostic charts allowed us to assess the behavior of the models at different training set sizes and hyperparameter values. They demonstrated that the chosen models strike a good bias-variance trade-off.

The ML model predicts the DLVO total interaction potential, *V*_t_, rather than treating CSC or critical pH as isolated empirical labels. CSC and critical pH are obtained by applying a threshold-extraction procedure to the predicted interaction potential surface.^56–58^ For a fixed sandstone–brine scenario, the trained surrogate predicts *V*_t_(*x*,*h*), where x denotes ionic strength or salinity for CSC prediction, pH for critical pH prediction, and h the particle-surface separation distance.^57,59^ For each candidate value of *x*, the surrogate predicts the total potential over the prescribed h-grid. This serves as a stability indicator, where the critical condition is identified as the value of *x* at which the total potential changes from attachment-favorable to detachment-favorable behavior, expressed as *V*_t_ (x_threshold)_Max = 0.^57,59^

RMSE and MAE quantify error in the predicted total interaction potential, *V*_t_, and are expressed the same way. They are not direct errors in the pH or salinity. Because the DLVO-generated *V*_t_ values span from strongly attractive negative values of approximately −500 kBT to positive repulsive values, RMSE and MAE values close to or below 1 kBT represent very small global errors relative to the total output range.^57,58^

The operational relevance of these errors is governed by the threshold region where the DLVO stability indicator crosses zero or satisfies the specified detachment criterion.^57,58^ A small global *V*_t_ error can produce either a small or a large threshold displacement, depending on the local value near the crossing. Therefore, minimized errors are mandatory to ensure accurate and precise predictions of both the CSC and pH values.

## 3 Results and Discussion

### 3.1 Exploratory Data Analysis and Response Distributions

Before developing the models, an exploratory data analysis (EDA) was conducted to evaluate the structure of the fines-migration data set and to understand the relationships between the input parameters and the variable output, namely, the interaction potential. This step is essential to verify data balance and distribution, identify outliers, study the general trends, and detect any multicollinearity that could influence the model stability. Figure 2 presents the count distributions of the key input features and target variable for both the pH data set (Figure 2a) and the salt concentration data set (Figure 2b). These figures reveal structural characteristics of the data set that directly affect data preprocessing and the selected model hyperparameter optimization.

**Figure 2:**
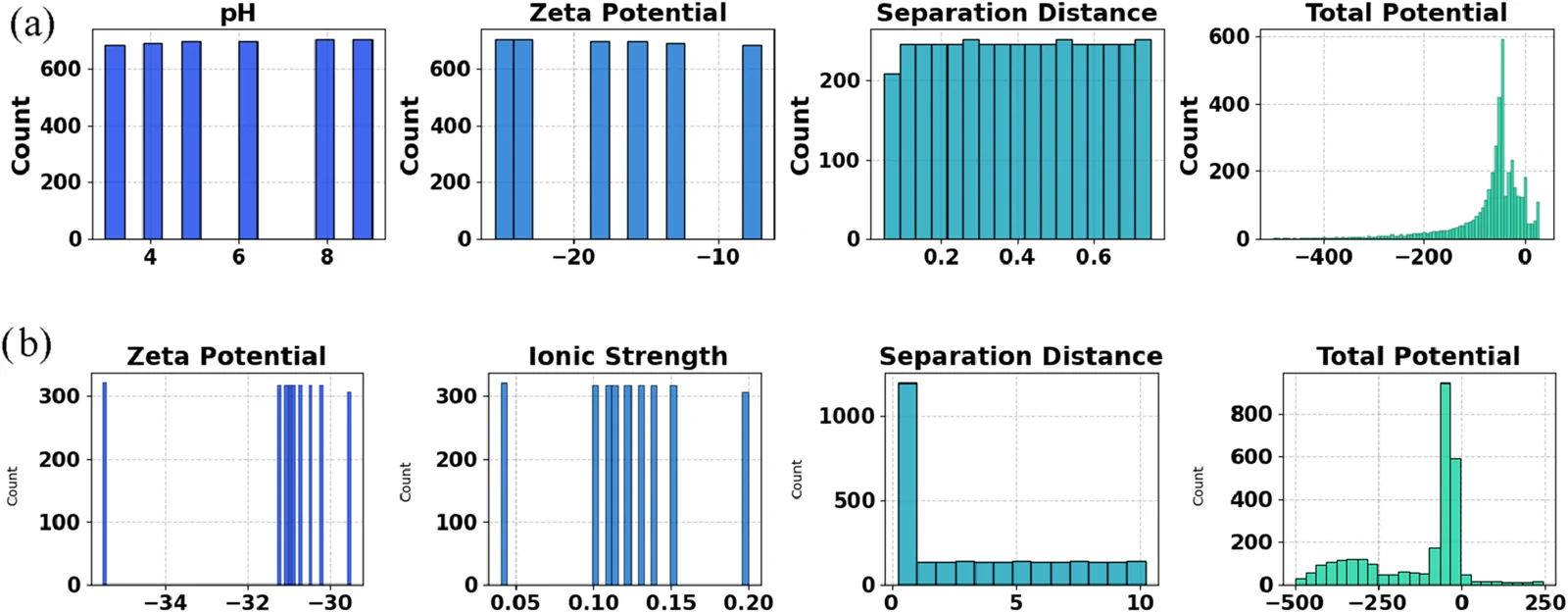
Count distributions of key input features and target variables for (a) the pH data set and (b) ionic-strength data set used in the fines-migration machine-learning framework.

In the pH data set, the count distributions of pH, zeta potential, and separation-distance counts remain consistent and are approximately uniform across their ranges, due to the grid-based DLVO calculations in which each pH level and separation-distance increment were sampled with equal density. This balanced coverage across input space is a favorable property for supervised learning, as it prevents the model from developing a statistical bias toward any particular operating condition and ensures that the learned function is well-constrained across the full physicochemical envelope relevant to fines migration.^42^ In contrast, the total potential distribution for the pH data set is strongly right-skewed, with the majority of observations concentrated at moderately negative values (approximately −100 to 0 kT) and a long tail extending toward −500 kT. This skewness directly reflects the underlying DLVO physics, strongly attractive conditions at low pH and small separations generate a disproportionately large number of deeply negative potential values, and is therefore physically meaningful rather than a sampling artifact.^29,60,61^

In the salt concentration data set, the distribution of separation distance is markedly nonuniform, exhibiting a pronounced peak at small separation values (near-zero) and a rapid decay toward larger distances; this pattern is inherent to the DLVO energy profile, wherein the interaction energy changes most rapidly at close approach and the physical system spends comparatively little time at large separations under realistic reservoir conditions. The ionic strength and zeta potential show discrete values of concentration levels, again reflecting the structured design. The total potential distribution for the ionic-strength data set has two clear maxima separated by a valley, with a dominant peak near zero and a secondary distribution extending to approximately −500 kT, consistent with the coexistence of near-neutral and strongly attractive interaction regimes.^5^

The dominance of strongly negative values relative to near-zero or positive values distribution of total potential poses a potential model bias. This is because ensemble learners trained on imbalanced data may optimize the performance in high-density regions while exhibiting weaker performance on the rest of the data set, which in this case is the high-positive-potential regime that corresponds precisely to the fines-mobilizing conditions of greatest practical interest.^62^ This issue was eliminated using three strategies: (i) the 10-fold cross-validation ensured that all regions of the target distribution were systematically represented in each validation fold and (ii) the use of several error metrics and plots during model evaluation ensured that large errors in physically critical regions of the target distribution were not masked by the dominant negative population.^53^

### 3.2 Feature Distributions and Pairwise Relationships in the Ionic-Strength Data Set

The pair plot, shown in Figure 3, presents the distribution and pairwise relationships between zeta potential, ionic strength, separation distance, and total potential for the salt concentration data set, and reveals several features that directly informed the feature engineering and modeling decisions in this study. The most important observation is the strong collinearity between zeta potential and ionic strength in an almost linear fashion. This relationship is physically well-founded, as increasing electrolyte concentration compresses the electrical double layer in accordance with the inverse square-root dependence of the Debye length on ionic strength (κ^–1^ ∝ *I* ^–1/2^), causing a reduction in the magnitude of the negative surface potential and driving zeta potential toward less negative values.^63–65^ Therefore, both variables encode the same underlying information and retaining both simultaneously introduces redundancy and can destabilize model estimates.^42^ Finally, the scatter panel relating separation distance to total potential reveals the characteristic nonmonotonic DLVO energy profile, where at small separations, van der Waals attraction dominates and drives total potential to strongly negative values, whereas at intermediate separations EDL repulsion can generate a local energy maximum (energy barrier) before secondary attraction reemerges at larger distances.^28,29,60^

**Figure 3:**
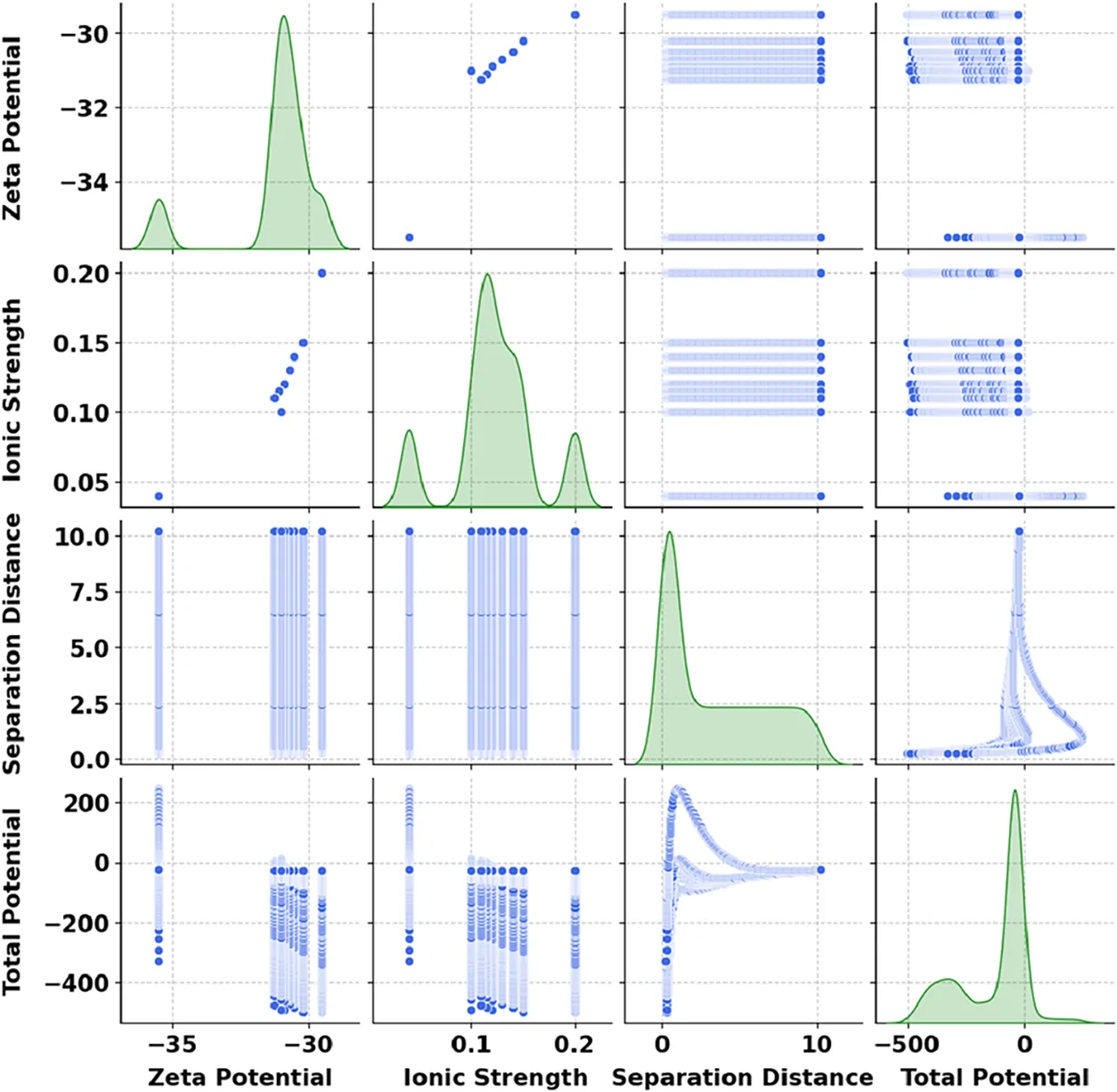
Pair plot of key input features and target variable for the ionic-strength data set.

### 3.3 Feature Distributions and Pairwise Relationships in the pH Data Set

The pair plot for the pH data set is shown in Figure 4. It reveals a strong negative, linear relationship between pH and zeta potential. This pH–zeta potential coupling is similar to the ionic-strength–zeta potential collinearity observed in the salinity data set. The plot of separation distance versus total potential reproduces the hallmark nonmonotonic DLVO energy curve where total potential rises from strongly negative values at close approach, dominated by van der Waals attraction, through an energy maximum at intermediate separations, before converging toward zero at large distances.^29,60,61^ Critically, the family of curves visible in this panel are due to changing pH levels, with high-pH conditions producing shallower energy minima, while low-pH curves show deep primary minima consistent with strong particle retention.^12,66^

**Figure 4:**
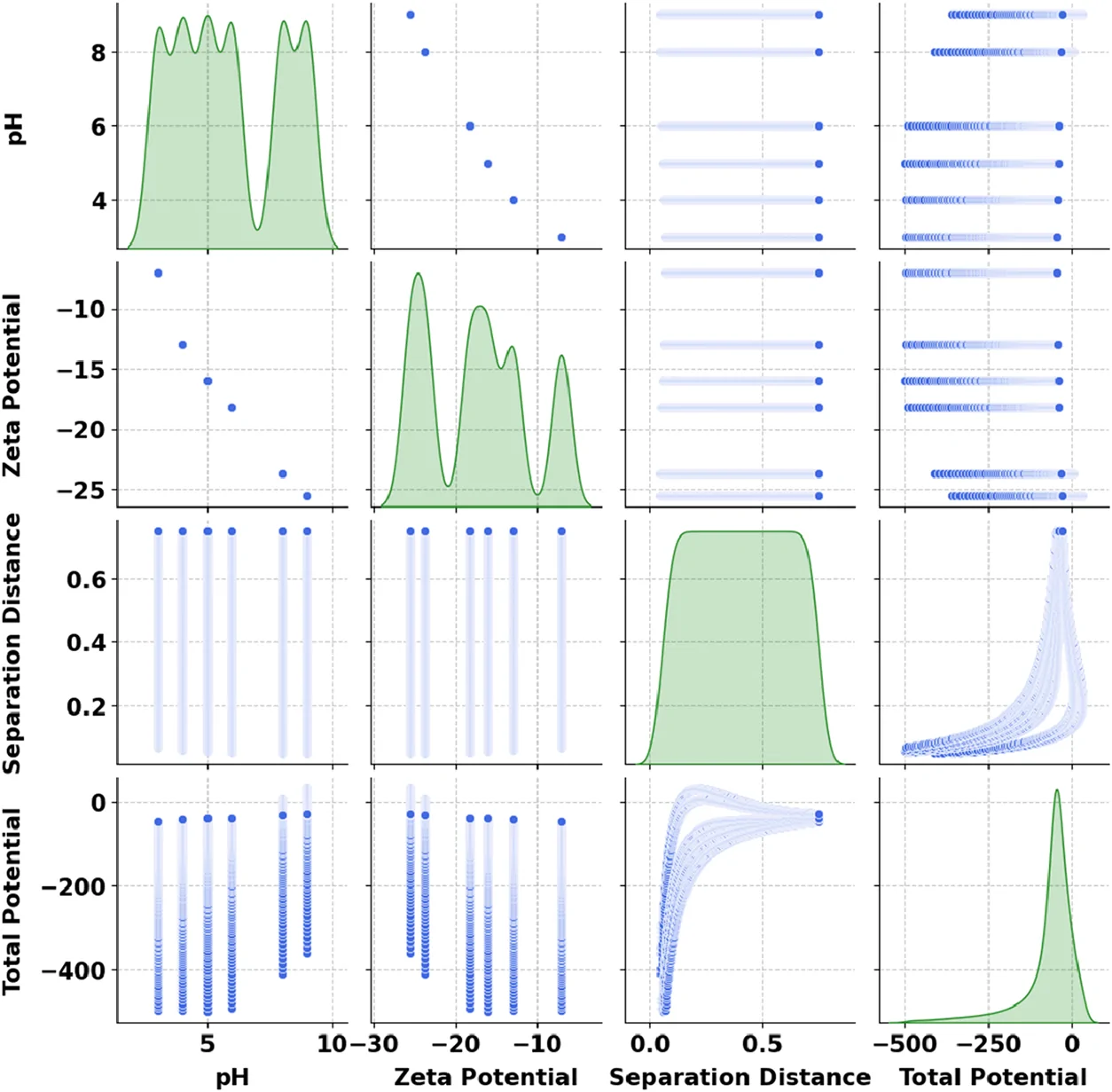
Pair plot of key input features and target variable for the pH data set.

### 3.4 Correlation Matrix Analysis of pH

The Pearson correlation matrix in Figure 5 shows extremely high correlation between pH and zeta potential (*r* ≈ −0.98), confirming that pH and zeta potential in this experiment describe the same underlying mechanism. Also, there is a moderate positive correlation between pH and total interaction potential (*r* ≈ 0.44), where pH strongly controls electrostatic surface behavior and energy-barrier evolution. On the other hand, separation distance shows a moderate positive correlation with total potential (*r* ≈ 0.52), indicating that geometric effects contribute independently to fines stability. Zeta potential was removed from this data set in favor of pH for two main reasons: first, to avoid multicollinearity and ensure model stability; and second, because pH can be easily measured under field and laboratory conditions using standard instrumentation, whereas zeta potential requires specialized electrokinetic equipment (such as electrophoretic light scattering or streaming-potential apparatus).^32,67^

**Figure 5:**
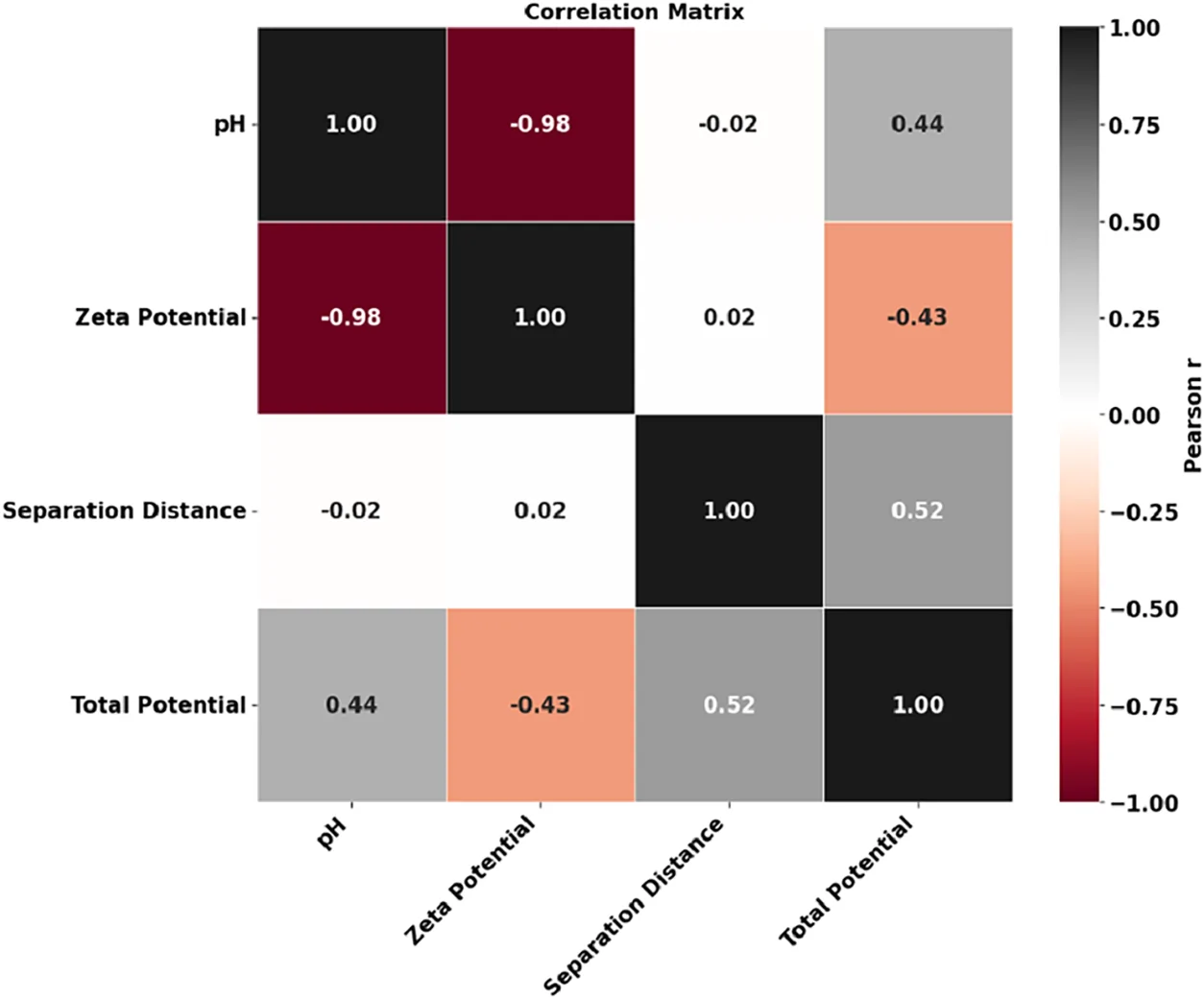
Heatmap of Pearson correlation coefficients for the main fines-migration parameters in the pH data set.

### 3.5 Correlation Matrix Analysis of Salts Concentration

Figure 6 presents the Pearson correlation matrix for the varied parameters in the ionic data set, namely, zeta potential, ionic strength, separation distance, and total interaction potential. The matrix shows a very strong positive correlation between zeta potential and ionic strength (*r* = 0.90), reflecting multicollinearity. In addition, there is a high positive correlation between separation distance and total interaction potential (*r* = 0.60), showing that larger particle separations are associated with a higher tendency for fines detachment. On the other hand, zeta potential and ionic strength both show moderate negative correlations with total interaction potential (*r* = −0.32 and *r* = −0.28, respectively), consistent with the interpretation that increased salinity and altered surface electrostatics reduce repulsive interactions and may favor fines mobilization. Because zeta potential and ionic strength were highly collinear (*r* = 0.90), zeta potential was excluded from the final modeling for the same reasons as in the pH data set. The correlation results provide a quantitative assessment of parameter sensitivity. For the pH data set, separation distance showed a stronger linear association with total interaction potential (*r* ≈ 0.52) than pH (*r* ≈ 0.44). Similarly, for the ionic-strength data set, separation distance exhibited the strongest association with total interaction potential (r ≈ 0.60), compared with ionic strength (*r* ≈ −0.28). These results indicate that separation distance strongly controls the magnitude and shape of the DLVO interaction-energy profile, whereas pH and ionic strength govern changes in electrostatic conditions and the location of the attraction-to-repulsion transition. The reported correlation coefficients represent linear sensitivity, while the nonlinear effects were evaluated through the ensemble-model comparisons, validation curves, and independent test results.

**Figure 6:**
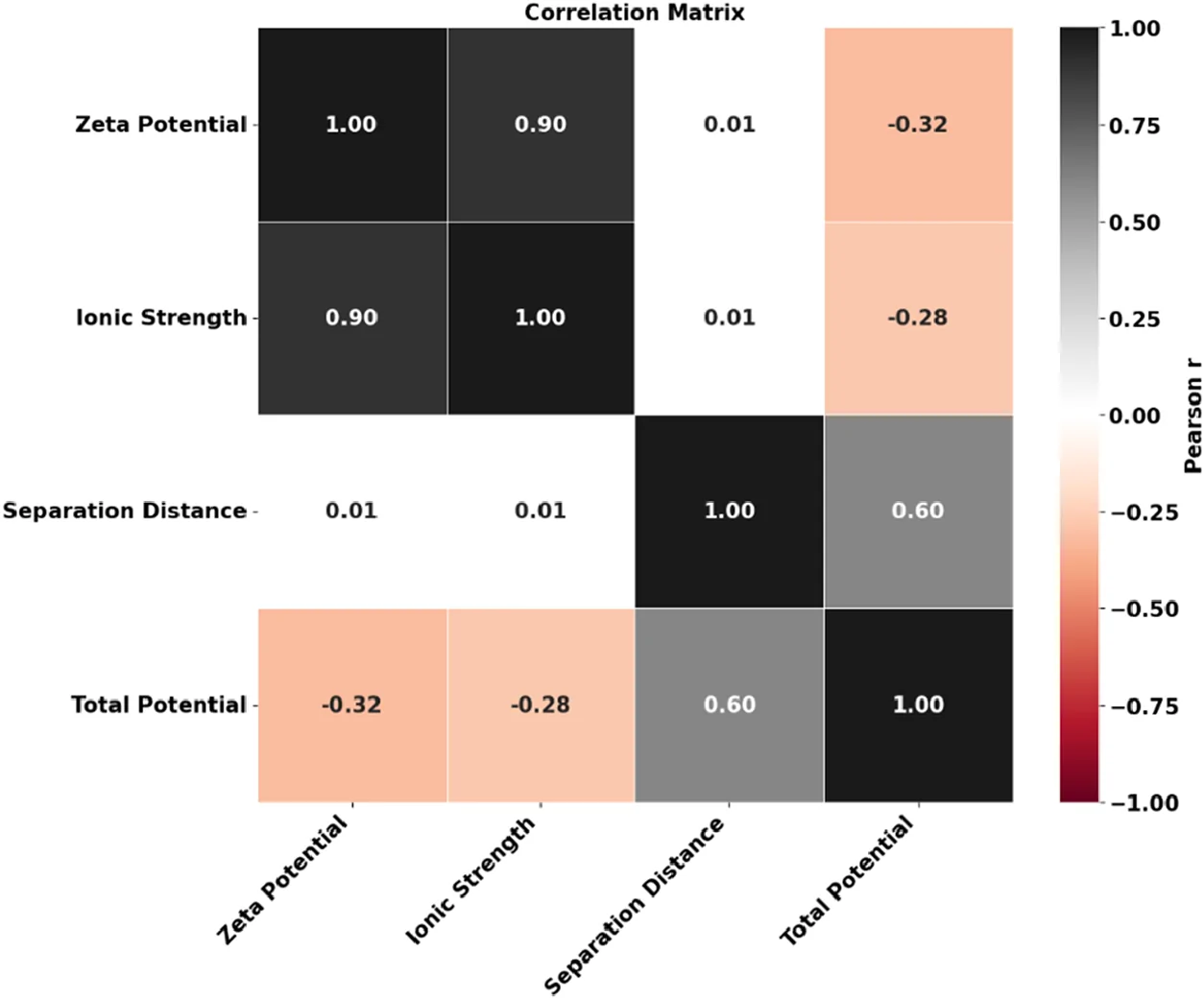
Heatmap of Pearson correlation coefficients for the main fines-migration parameters in the ionic-strength data set.

### 3.6 Performance of Ensemble Models

To assess and compare performance of the ensemble, both quantitative metrics (*R* ^2^, RMSE, and MAE) and graphical analyses (predicted vs actual plots and residual distributions) were used for the training and unseen test data sets. This dual evaluation enables model accuracy quantification and systematic bias or trend identification.

#### 3.6.1 Comparative Performance Overview of pH

##### 3.6.1.1 Initial Results

In the DLVO formulation, the total interaction potential, *V*_t_, arises from the balance among London–van der Waals attraction, electrostatic double-layer repulsion, and short-range Born repulsion. Changing pH modifies protonation and deprotonation equilibria at quartz and clay-mineral surface sites, thereby changing the magnitude of surface charge and the corresponding electrostatic repulsive term. Under acidic to near-neutral conditions, reduced negative surface charge weakens double-layer repulsion and deepens the primary attractive minimum, favoring fines retention. Under more alkaline conditions, deprotonation increases negative surface charge and strengthens electrostatic repulsion, moving the *V*_t_ profile toward the repulsion-dominant state used to identify the critical pH. The steepness of this transition explains why the pH target is physically nonlinear even though the input space contains only a small number of operational descriptors.^57–59,68^

As pH increases, the total interaction potential changes from net attractive to net repulsive, and at that point particle detachment is initiated.^9,69^ This transition is abrupt and highly nonlinear, where small changes in pH near the critical value can produce disproportionately large changes in the total interaction energy. At this range, the response is steep and difficult to approximate with low-complexity ML models. Due to this, nine ensemble models were evaluated on both training and independent test sets, and the results are summarized in Table 2.

**Table 2:** Performance Metrics of Ensemble Models for Fines-Migration Prediction Using pH

model	*R* ^2^ (train)	RMSE (train)	MAE (train)	*R* ^2^ (test)	RMSE (test)	MAE (test)
Extra Trees	1.0000	≈0	≈0	0.9999	0.54	0.10
Random Forest	0.9999	0.80	0.19	0.9992	2.48	0.65
Bagging Trees	0.9998	1.06	0.24	0.9991	2.78	0.73
Extra Gradient Boosting	0.9994	1.96	0.57	0.9988	3.10	1.03
CatBoost	0.9994	1.96	0.63	0.9989	2.95	0.94
LightGBM	0.9977	3.92	0.96	0.9976	4.41	1.36
Gradient Boosting	0.9962	4.97	3.70	0.9963	5.51	4.09
XGBRF	0.9908	7.77	5.03	0.9916	8.30	5.58
AdaBoost	0.9278	21.74	17.99	0.9378	22.64	18.33

The evaluation revealed a clear performance distinction. At the lower end, AdaBoost performed poorly with a test *R* ^2^ of only 0.9378 and a test MAE of 18.332, demonstrating that its sequential weak-learner architecture, which incrementally corrects residuals through reweighted sampling, lacks the structural depth in this case. These errors are unacceptable for a model intended to identify the precise pH threshold at which fines mobilize, as such error of this magnitude could lead to significant misclassification of the stability state of the fine–grain system. XGBRF performed marginally better (test *R* ^2^ = 0.9916, RMSE = 8.30), yet its hybrid boosting and random forest architecture still showed a scatter at the extremes of the response range, indicating residual difficulties. Gradient Boosting and LightGBM, on the other hand, improved progressively with a test *R* ^2^ = 0.9963 and 0.9976, respectively, with declining RMSE values reflecting the increasing capacity of deeper, leaf-wise tree growth. However, their MAE values of 4.09 and 1.36, respectively, remained nonnegligible. CatBoost, Extra Gradient Boosting, and Bagging Trees all achieved test *R* ^2^ values above 0.999 with RMSE below 3.2, which is excellent, confirming that sufficiently deep ensemble architectures with robust regularization can capture this phenomenon better. Their performance differences are attributable to the distinct learning strategies (boosting with ordered target statistics versus variance-averaging over bootstrap samples) and their differing sensitivity to the sharp nonlinear boundaries in the data set. Random Forest further improved on these results (test *R* ^2^ = 0.9992, RMSE = 2.48, MAE = 0.65), benefiting from its random subspace sampling. The Extra Trees regressor achieved the highest predictive accuracy among all evaluated algorithms, with a test *R* ^2^ of 0.9999, RMSE of 0.54, and MAE of only 0.10. This exceptional result is due to its randomized split-point selection strategy, which introduces additional variance reduction relative to standard Random Forest by eliminating the greedy optimization of split thresholds, thereby producing a smoother and more generalizable approximation of the highly nonlinear pH–total potential response surface.^48^ The near-zero training RMSE and MAE for Extra Trees shows the model’s full interpolation capacity on the training set, with an absence of overfitting when tested on unseen data.

The observed model hierarchy is therefore consistent with the shape of the pH-controlled DLVO response surface. The data are not governed by a globally linear relation between pH and V_t_; instead, they form a piecewise nonlinear surface in which the separation distance controls the geometry of the energy profile, while pH controls the electrostatic contribution through surface-charge regulation. At nanometer-scale separation, van der Waals attraction and Born repulsion vary rapidly with h, whereas the electrostatic term changes with pH-dependent surface potential and screening length. Consequently, the target contains regions of slow variation, steep near-threshold curvature, and strongly attractive negative-potential states. Tree ensembles are well-suited to this type of tabular physics-derived response because they approximate nonlinear surfaces by partitioning the feature space into locally homogeneous regions rather than imposing a single global functional form.^58,68,70,71^

##### 3.6.1.2 Predicted vs Actual Analysis

Predicted–actual parity plots show that the Extra Trees, CatBoost, and Random Forest models align almost perfectly with the 1:1 reference line across both training and test sets shown in Figure 7. This is a direct indicator of the model’s predictive capabilities. In contrast, AdaBoost exhibit broader scatter and bias, consistent with their low *R* ^2^ and higher error metrics.

**Figure 7:**
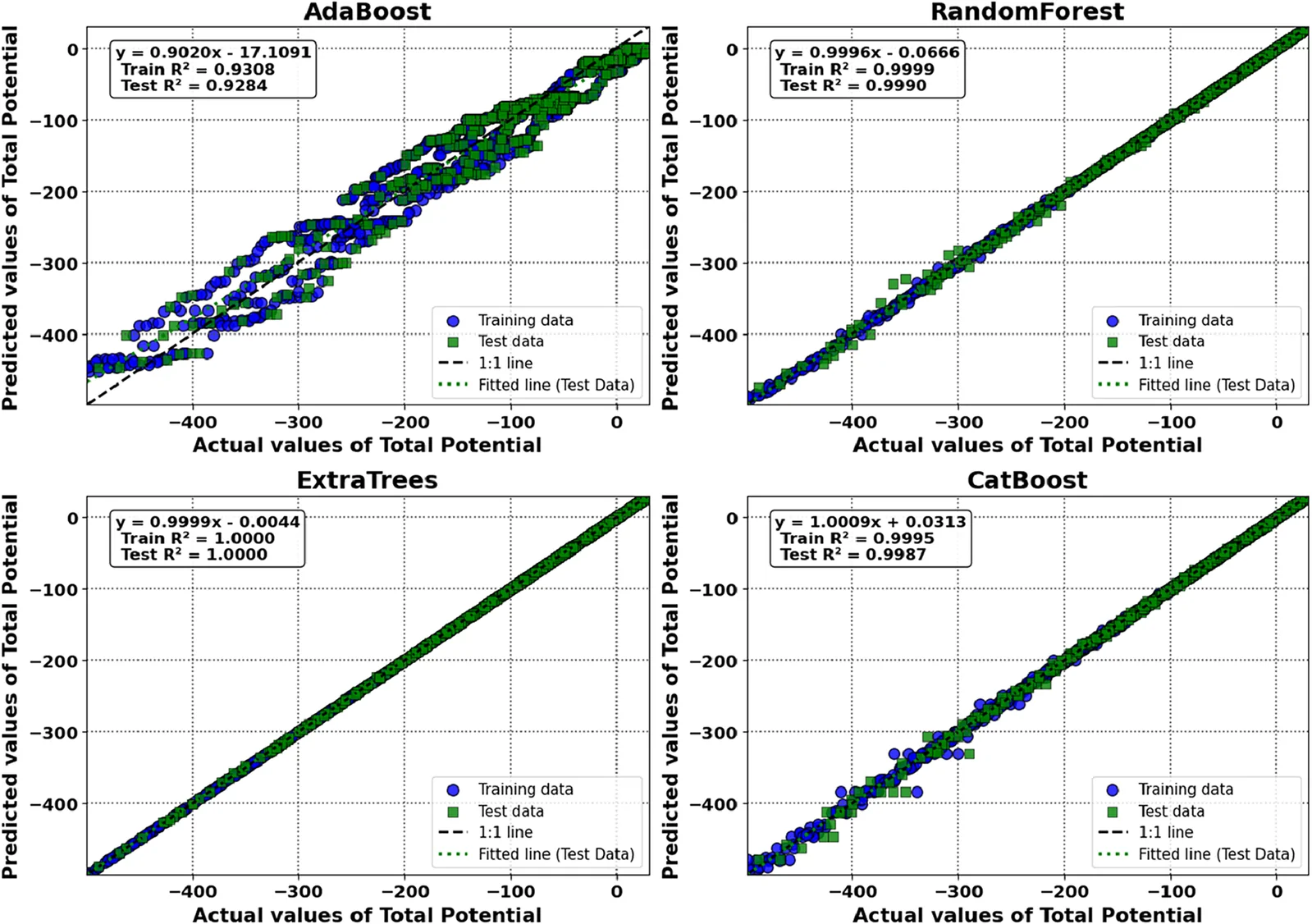
Comparison of predicted and actual fines-migration responses for selected ensemble models on training and test data for pH.

##### 3.6.1.3 Residual Analysis

Figure 8 demonstrates examples of residual plots for the tested models. It provides further confirmation of the model reliability and bias in highly populated zones. For the best-performing models, residuals are symmetrically distributed around zero with no visible heteroscedasticity, indicating unbiased predictions and strong generalization. Lower performing models show structured residual patterns.

**Figure 8:**
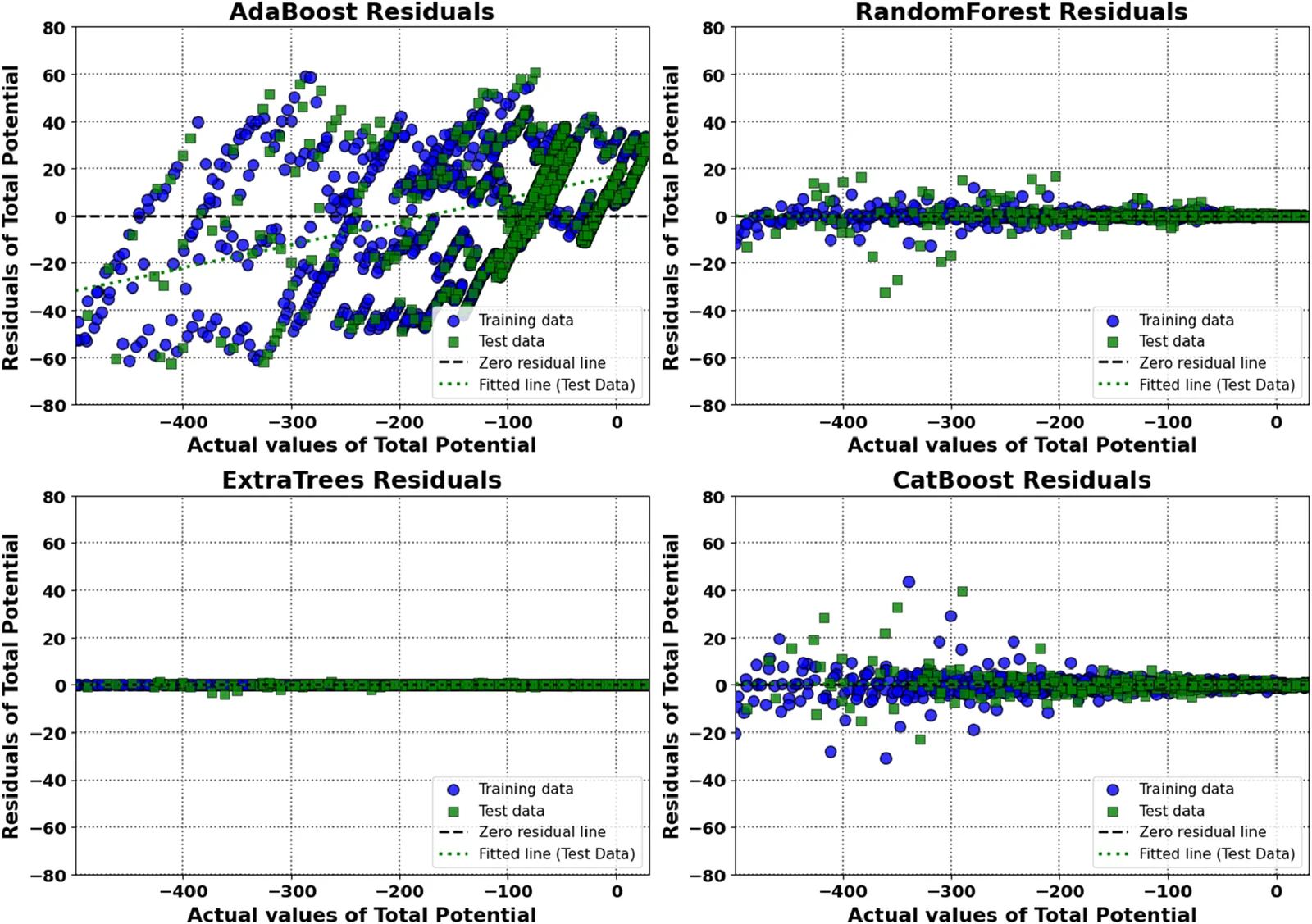
Residual plots for training and test data sets of selected ensemble models used for pH fines-migration prediction.

##### 3.6.1.4 Optimized Extra Trees Model

Hyperparameter optimization of the Extra Trees model for the pH data set was first guided by validation curves for max_depth, n_estimators, and learning curve analysis. The n_estimators validation curve in Figure 9a, demonstrates that training and CV *R* ^2^ are indistinguishable across the full range, above 0.999, confirming that n_estimators can be set conservatively without sacrificing predictive accuracy. The max_depth validation curve illustrated in Figure 9b shows CV *R* ^2^ rising steeply from approximately 0.38 at max_depth = 1 to 0.65 at depth 3, 0.82 at depth 4, and 0.96 at depth 5 before reaching its maximum value (*R* ^2^ ≥ 0.99) at max_depth = 10. Finally, the learning curve in Figure 9c illustrates that CV *R* ^2^ starts at approximately 0.993 at the smallest training size (∼230 samples) and converges toward unity, with the training and CV gap effectively closing after approximately 900 samples. This is because the data set has become large enough to achieve full convergence on the wider pH range and greater diversity of electrostatic states.

**Figure 9:**
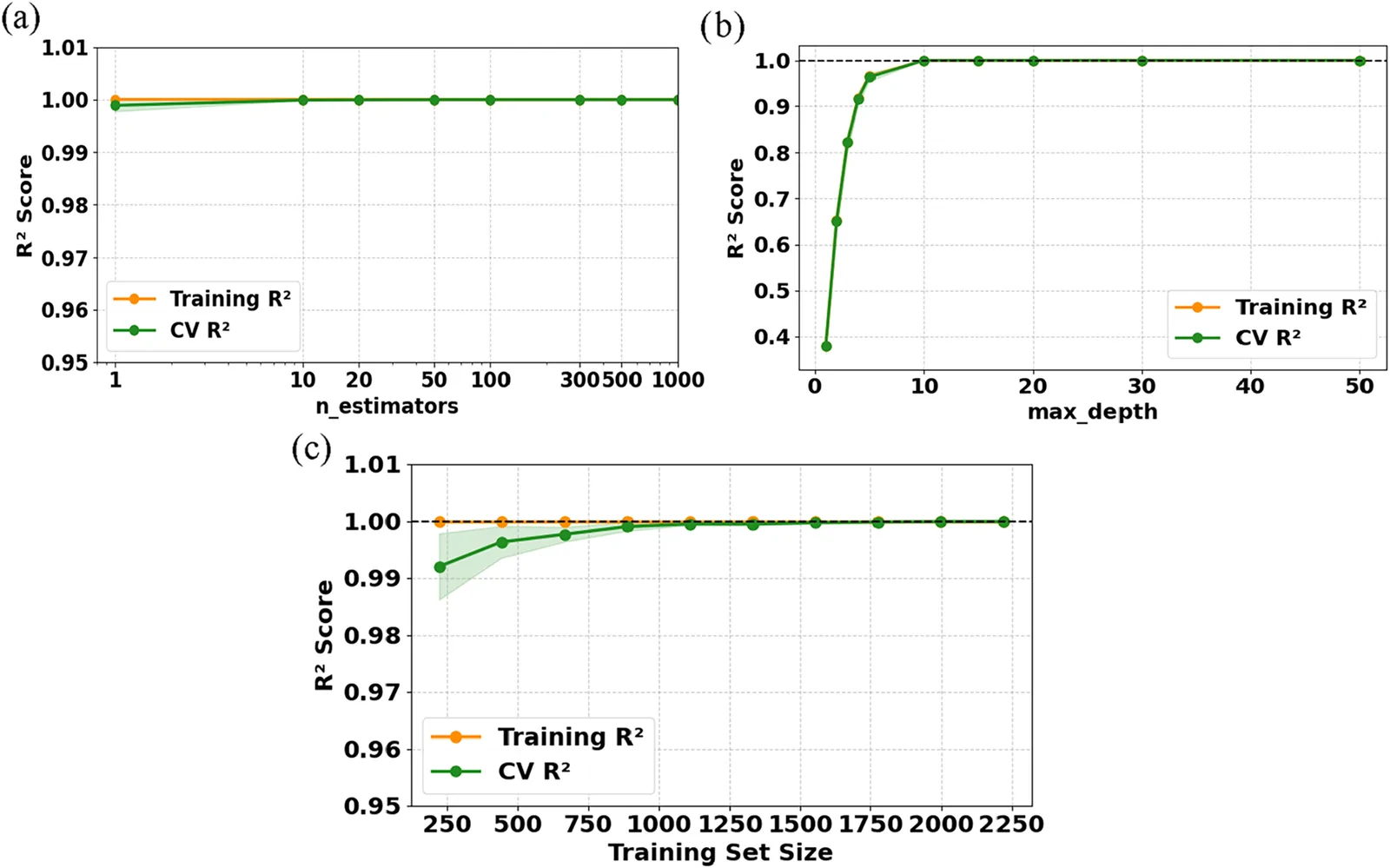
Validation curve for (a) n_estimators, (b) max_depth, and (c) training set size for the optimized Extra Trees pH model.

To further enhance predictive performance, the Extra Trees Regressor was optimized using Bayesian hyperparameter tuning with Optuna. The optimization was conducted using a tree-structured Parzen estimator (TPE) sampler with a 10-fold cross-validation. The objective was to minimize the RMSE while preventing overfitting. The final optimized hyperparameters were

n_estimators=1000,max⁡_depth=None,min⁡_samples_split=2,min⁡_samples_leaf=1.

The optimized Extra Trees model demonstrated exceptional predictive accuracy, where the training set: *R* ^2^ ≈ 1.000, RMSE ≈ 1.35 × 10^–14^, MAE ≈ 7.70 × 10^–15^, the cross-validation mean *R* ^2^ ≈ 0.999927, RMSE ≈ 0.70, MAE ≈ 0.11, and the test set *R* ^2^ ≈ 0.999998, RMSE ≈ 0.12, MAE ≈ 0.04. Predicted vs actual, residual plots, and residual distribution plots shown in Figure 10 confirm near-perfect alignment along the parity line, with residuals being symmetrically distributed near zero. The distribution of residuals is symmetrical and even tighter around the zero line on the residuals plot indicating no bias behavior. Also, the consistency of the model on train, CV, and test confirm that there is no overfitting present.

**Figure 10:**
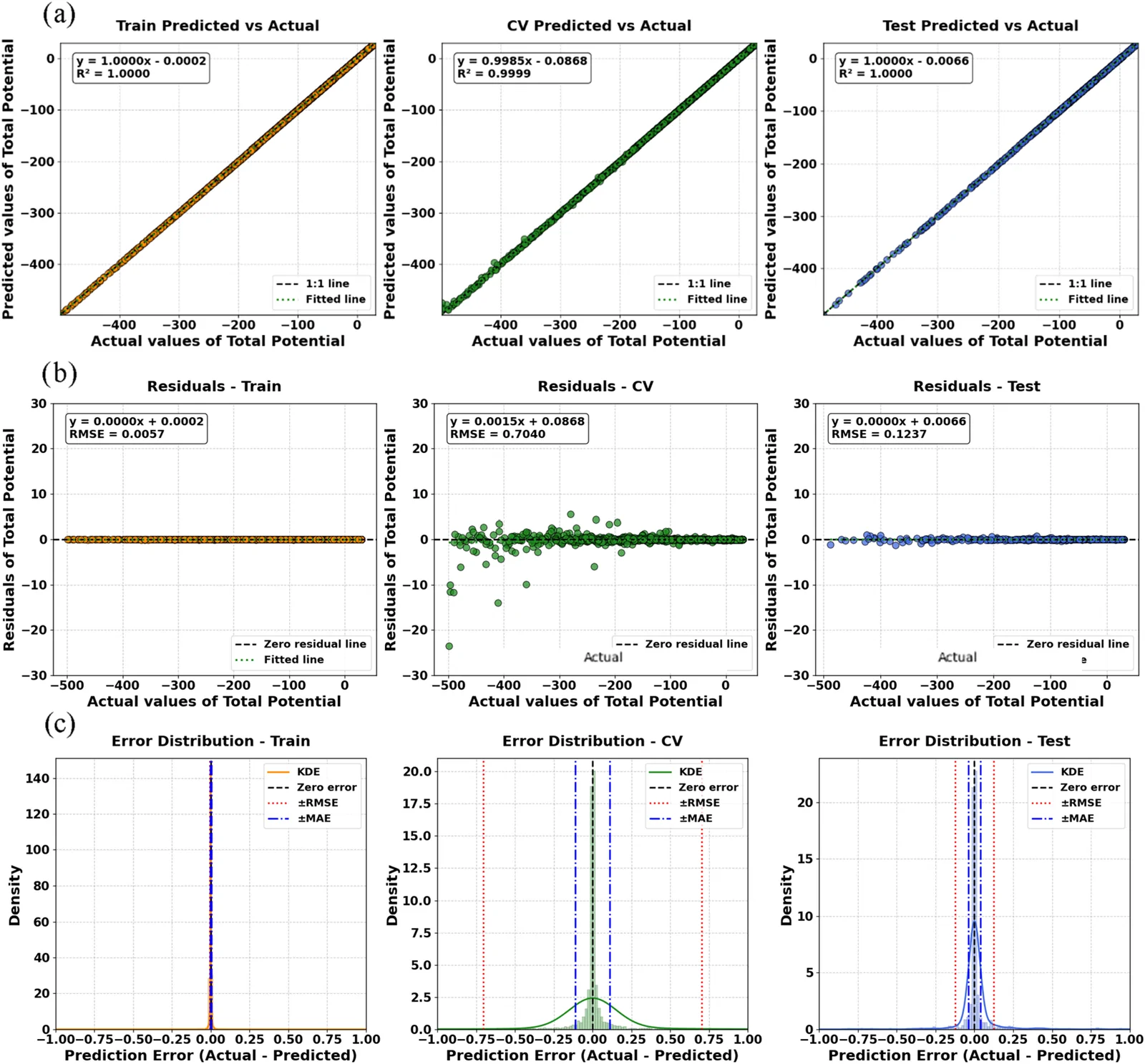
(a) Predicted versus actual, (b) residual plots, and (c) error distribution for training, cross-validation, and test data sets for the optimized Extra Trees model used for pH fines-migration prediction.

The reduction in the pH-model RMSE from the baseline Extra Trees value of approximately 0.54 kBT to the optimized value of approximately 0.12 kBT is statistically measurable. Both models, however, already reconstruct the DLVO potential surface with high accuracy relative to the full *V*_t_ range.^56,57^ The practical value of Bayesian optimization is therefore best interpreted as an incremental refinement rather than a fundamental change in model capability.

This refinement is most relevant near the threshold zone, where small deviations in *V*_t_ can shift the interpolated critical pH if the local slope of the stability indicator is modest.^58,72^ Optuna tuning therefore improves numerical robustness and threshold localization while confirming that the baseline Extra Trees architecture was already close to its effective operating optimum for the present DLVO-derived data set.^71^

The 10-fold cross-validation results, demonstrated in Figure 11, for the optimized Extra Trees pH model demonstrate exceptional and uniform predictive performance across all data partitions, with six of ten folds attaining a perfect *R* ^2^ of 1.00000 and the remaining folds yielding values no lower than 0.99972 (Fold 8), resulting in a mean CV *R* ^2^ of 0.9999. The near-zero interfold variance confirms that the model’s performance is consistent on all the subsets of the training data, satisfying the generalizability criterion required for a reliably deployed model.

**Figure 11:**
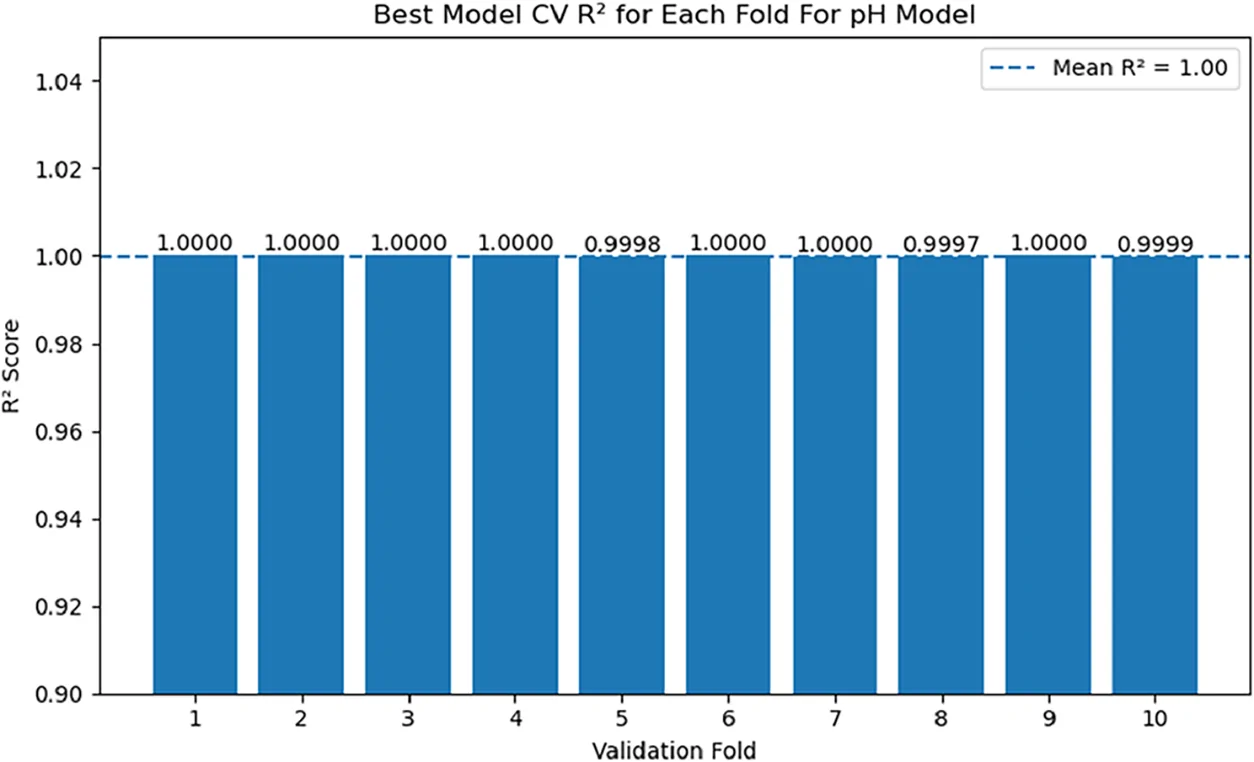
Cross-validation *R* ^2^ scores for the optimized Extra Trees model across all ten folds for the pH data set.

#### 3.6.2 Comparative Performance Overview of Critical Salt Concentration

##### 3.6.2.1 Initial Results

The CSC model represents an ionic-strength-controlled electrostatic screening problem. In DLVO theory, salinity affects the electrostatic double-layer term through the Debye screening length, so decreasing ionic strength expands the double layer and increases repulsive interaction, whereas increasing ionic strength compresses the double layer and promotes attachment-dominated energy profiles. This salinity dependence is coupled to the separation-distance coordinate because van der Waals attraction, Born repulsion, and electrostatic repulsion vary strongly across nanometer-scale values of h. The regression target is therefore a curved surface, V_t_(I,h), with its highest operational sensitivity concentrated near the critical salt concentration, where the predicted stability indicator changes sign or reaches the specified detachment criterion.^56–59,68^

The total interaction potential is characterized by a steep transition from net attractive (favoring particle retention) to net repulsive (triggering detachment and migration) as ionic strength changes above or below the critical salt concentration (CSC). This transition is a highly nonlinear, abrupt change in interaction energy across a narrow salinity interval, producing a steep response that represents a demanding regression target for data-driven.^12,32^ The nine ensemble models were again evaluated and tested on this task, and their results are summarized in Table 3.

**Table 3:** Performance Metrics of Ensemble Models for Fines-Migration Prediction for Ionic Strength

model	*R* ^2^ (train)	RMSE (Train)	MAE (train)	*R* ^2^ (test)	RMSE (test)	MAE (test)
Extra Trees	1.0000	≈0	≈0	0.99996	0.89	0.43
CatBoost	0.9999	1.50	0.67	0.9999	1.52	0.86
Random Forest	0.9999	1.06	0.41	0.9998	1.99	1.03
Bagging Trees	0.9999	1.36	0.60	0.9997	2.69	1.29
Extra Gradient Boosting	0.9999	1.36	0.57	0.9997	2.63	1.45
LightGBM	0.9994	3.71	1.45	0.9989	4.79	1.99
Gradient Boosting	0.9989	4.85	3.39	0.9985	5.77	3.93
XGBRF	0.9759	22.9	14.07	0.9708	25.08	15.04
AdaBoost	0.9705	25.33	19.85	0.9711	24.91	19.35

The performance evaluation produced similar results to that observed for the pH target, but with slightly higher absolute errors across most models. AdaBoost again produced the weakest predictions, with a test *R* ^2^ of 0.9711 and a test MAE of 19.346; the large residuals associated with this model confirm that its sequential, shallow-learner architecture is fundamentally inadequate for capturing the steep nonlinear dependence of interaction energy on ionic strength, particularly near the CSC transition where the response surface gradient is steepest.^73^ XGBRF exhibited a substantially more severe performance degradation, with a test *R* ^2^ of only 0.9708 and a test RMSE of 25.079 (the worst performance among all models for this target). On the other hand, Gradient Boosting improved considerably (test *R* ^2^ = 0.9985, RMSE = 5.76), and LightGBM further advanced performance (test *R* ^2^ = 0.9989, RMSE = 4.79, MAE = 1.99). This is a result of its leaf-wise tree growth enabling more precise resolution of the high-gradient regions of the ionic-strength–potential landscape. Extra Gradient Boosting and Bagging Trees both achieved excellent test *R* ^2^ values of 0.9997 with RMSE values below 2.7, again confirming that sufficiently deep ensemble architectures can approximate the dominant nonlinear variance in the CSC data set; however, their MAE values above 1.2 remained nonnegligible.^6,74^ Random Forest performed strongly (test *R* ^2^ = 0.9998, RMSE = 1.99, MAE = 1.03), and CatBoost matched this level with a test *R* ^2^ of 0.9999, RMSE of 1.52, and MAE of 0.86. At last, the Extra Trees regressor again achieved the highest predictive accuracy across all algorithms, with a test *R* ^2^ of 0.99996, RMSE of 0.89, and MAE of 0.43. Its superiority over all competing models can be attributed to its randomized split-point selection algorithm, which effectively suppresses overfitting while maintaining the flexibility to resolve sharp transitions at the CSC boundary. The negligible training test performance gap confirms that high accuracy was achieved without overfitting, satisfying the generalizability requirements.

The V_t_ response geometry explains why Extra Trees outperformed the other ensemble models for the salinity-dependent task. The stochastic split-threshold design of Extra Trees permits many alternative local partitions of the ionic-strength-separation space, and ensemble averaging then retains the stable components of those partitions while suppressing variance. Such behavior is advantageous for deterministic DLVO-generated tabular data in which the function is smooth overall but steep near CSC. Random Forest, Bagging, and CatBoost also resolve much of the nonlinear structure, but their dependence on bootstrap sampling, greedy split optimization, or sequential residual correction can leave slightly larger errors when the critical transition occupies a relatively narrow portion of the input domain. The weaker performance of AdaBoost and XGBRF is consistent with this interpretation because shallow residual correction or excessive averaging can reproduce the dominant attractive regime while smoothing the less populated near-zero and repulsive regions that control CSC extraction.^70,72,75–77^

##### 3.6.2.2 Predicted vs Actual Analysis

The predicted versus actual plots presented in Figure 12 visually confirm the quantitative performance of the ensemble models, where larger scatter (dispersion) around the 1:1 line indicates a poorer model performance. The Extra Trees, CatBoost, and Random Forest models exhibit very tight alignment along the ideal parity line for both training and test data sets. These models exhibit consistent predictions across the entire range of values, demonstrating excellent generalization and minimal bias. In contrast, AdaBoost shows noticeably greater scatter across the whole range in both train and test data sets. This deviation highlights the limited capacity.

**Figure 12:**
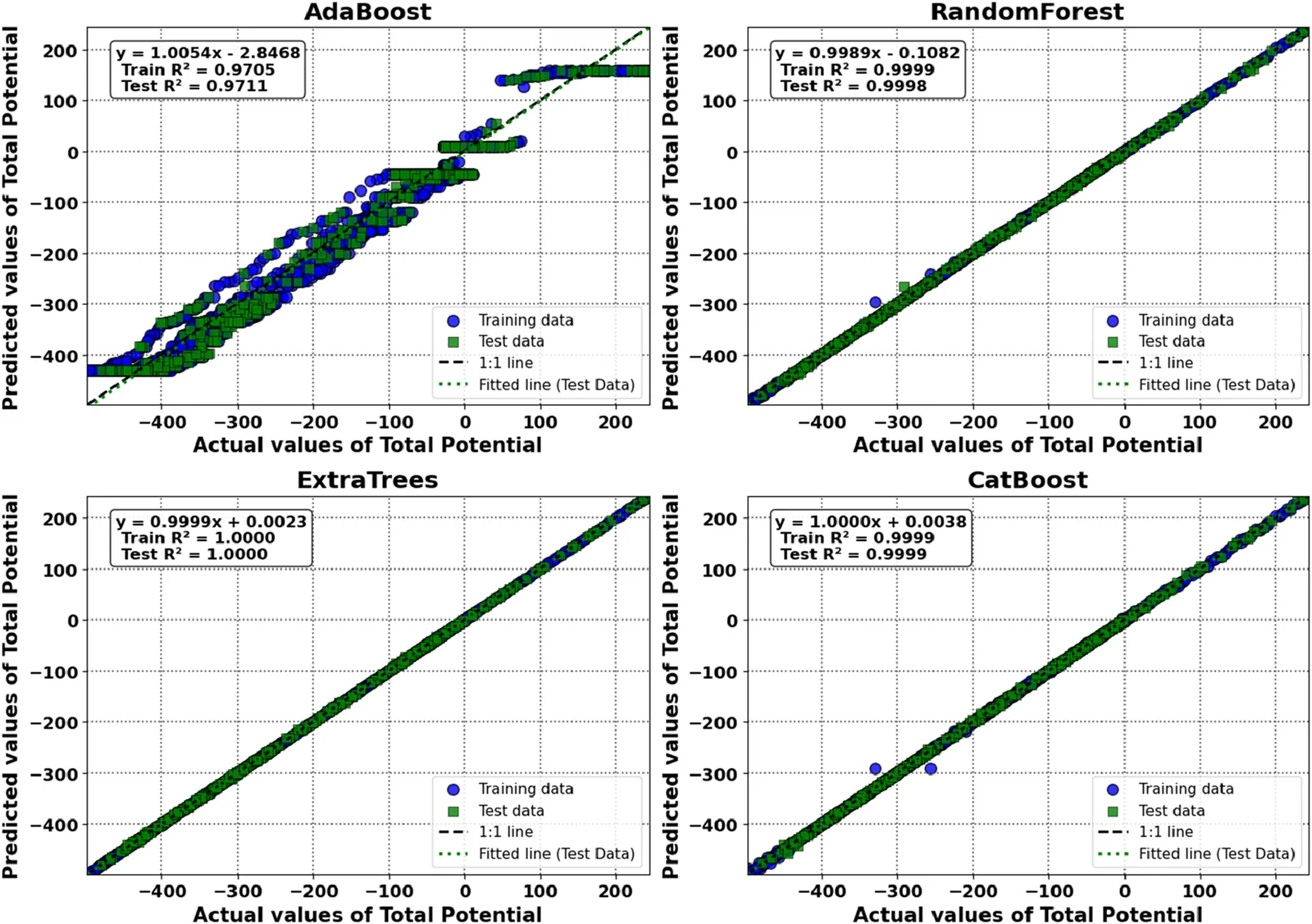
Comparison of predicted and actual fines-migration responses for selected ensemble models on training and test data for ionic strength.

##### 3.6.2.3 Residual Analysis

Residual analysis, demonstrated in Figure 13, is especially useful to detect bias. In this case, it further supports the robustness of the leading models. For the top-performing algorithms, the residuals are distributed approximately symmetrically around zero and show no obvious evidence of heteroscedasticity. In contrast, the weaker models exhibit an irregular residual structure, indicating a clear underfitting.

**Figure 13:**
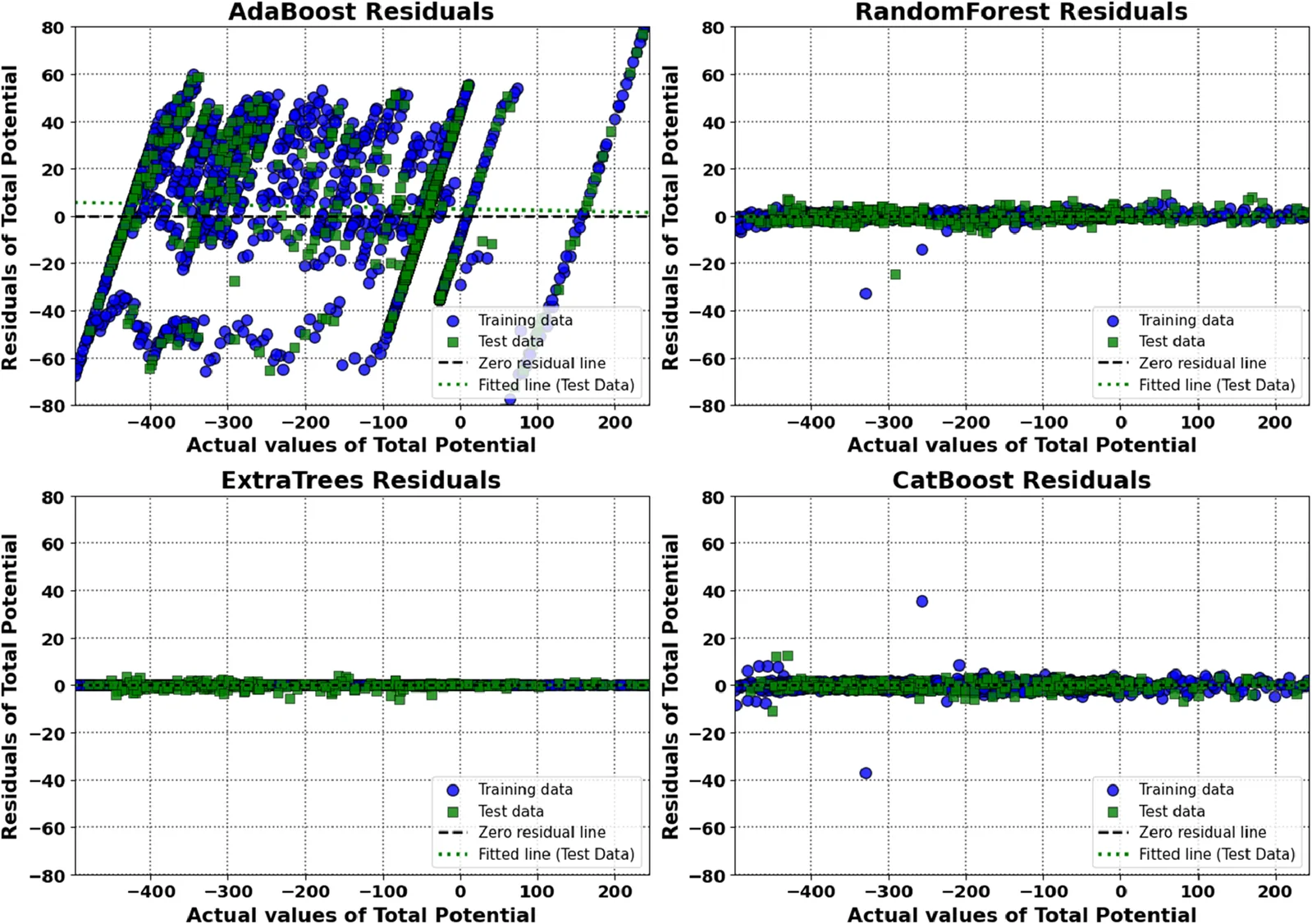
Residual plots for training and test data sets of selected ensemble models used for ionic-strength fines-migration prediction.

##### 3.6.2.4 Optimized Extra Trees Model

Prior to hyperparameter optimization of the Extra Trees model for the ionic-strength target, the effects of n_estimators, max_depth, and learning curve are demonstrated in Figure 14. The max_depth validation curve demonstrates a steep increase in CV *R* ^2^ from approximately 0.37 at max_depth = 1 to near-unity performance (*R* ^2^ ≥ 0.99) at max_depth = 10, beyond which both training and CV curves plateau and remain coincident up to max_depth = 50. The n_estimators validation curve demonstrates that the number of trees has minimal effect on overall performance of the model. The best CV *R* ^2^ score was selected for both hyperparameters. The learning curve highlights that CV *R* ^2^ increases rapidly from approximately 0.993 at the smallest training set size (∼175 samples) and converges to ≥ 0.9999 beyond approximately 1000 samples, the absence of a gap between training and CV performance at full data set scale confirms that the model operates in a data-sufficient regime free of residual overfitting.^53^

**Figure 14:**
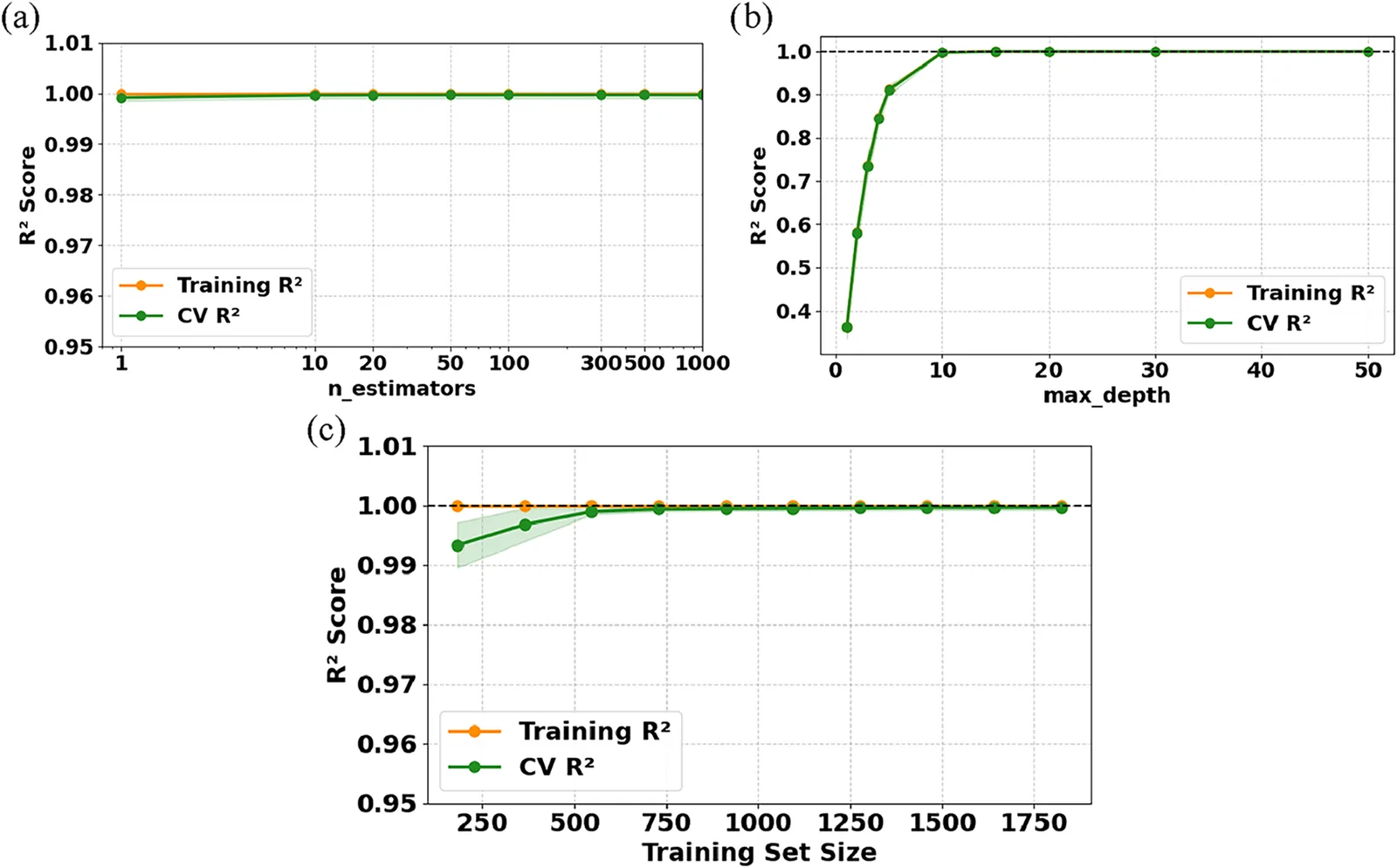
Validation curve for (a) n_estimators, (b) max_depth, and (c) training set size for the optimized Extra Trees ionic-strength model.

To further enhance predictive performance beyond the baseline ensemble models, an optimized Extra Trees Regressor was developed and fine-tuned using an extensive hyperparameter search. These settings were chosen by Bayesian optimization using the Optuna library hyperparameter tuning aimed at minimizing the validation error while preventing overfitting. The model was trained by using a 10-fold cross-validation scheme to ensure statistical robustness and generalization. The final optimized hyperparameters were

n_estimators=1000,max⁡_depth=None,min⁡_samples_split=2,min⁡_samples_leaf=1,max⁡_features=1.0,bootstrap=False

The optimized Extra Trees model demonstrated exceptional predictive accuracy, where the training set: *R* ^2^ ≈ 1.000, RMSE ≈ 1.35 × 10^–12^, MAE ≈ 8.27 × 10^–13^, cross-validation mean *R* ^2^ ≈ 0.99995, RMSE ≈ 0.99, MAE ≈ 0.42, and test set: *R* ^2^ ≈ 0.99997, RMSE ≈ 0.75, MAE ≈ 0.34. Predicted vs actual parity plots, shown in Figure 15a, for the training, cross-validation, and test splits collapse tightly onto the 1:1 line. The residuals are symmetrically distributed around zero without visible heteroscedasticity as illustrated in Figure 15b. The residual distributions, shown in Figure 15c, for training, cross-validation, and test sets are sharply centered around zero and exhibit narrow spreads, showing that most prediction errors are very small in magnitude across the entire range.

**Figure 15:**
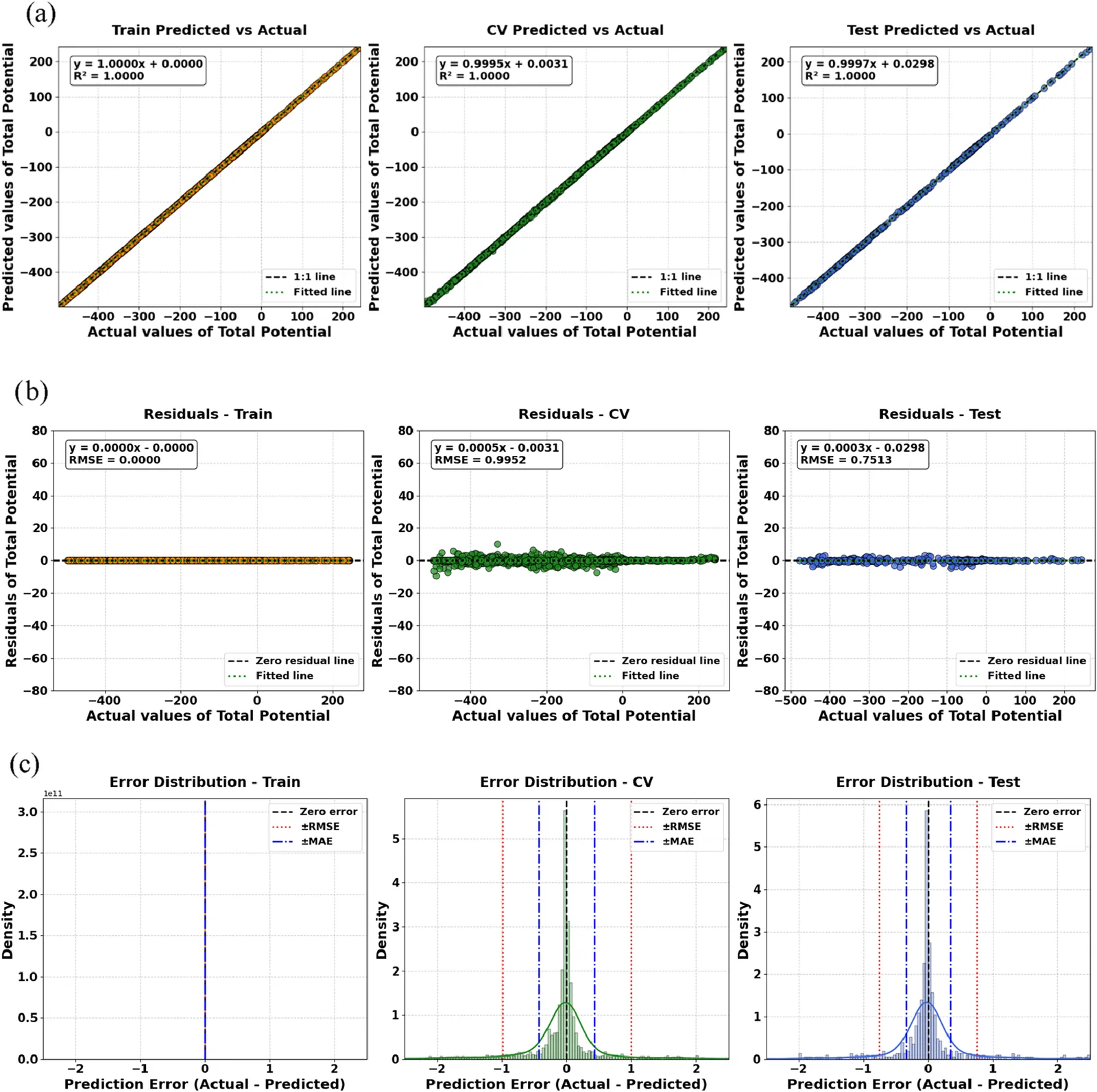
(a) Predicted versus actual, (b) residual plots, and (c) error distribution for training, cross-validation, and test data sets for the optimized Extra Trees model used for ionic-strength fines-migration prediction.

Similar to the pH Extra Trees optimized model, the CSC optimized model reduces RMSE from the baseline Extra Trees value of 0.9–0.75 kBT, a slight improvement when compared to the range of total potential, but most relevant near the threshold zone.^58,72^

Also, shown in Figure 16 are the 10-fold cross-validation results for the optimized Extra Trees model, which demonstrate highly consistent predictive performance across all data partitions, with per-fold *R* ^2^ values ranging from 0.99887 (Fold 2) to 0.99997 (Fold 9) and a mean CV *R* ^2^ of 0.9998. The negligible interfold variance confirms that the model generalizes uniformly across all the subsets of the data set and is not sensitive to any data partition. The near-unity *R* ^2^ sustained across all ten folds, without a single fold falling below 0.998, indicating that the training set size is large enough relative to the complexity of the learned function.

**Figure 16:**
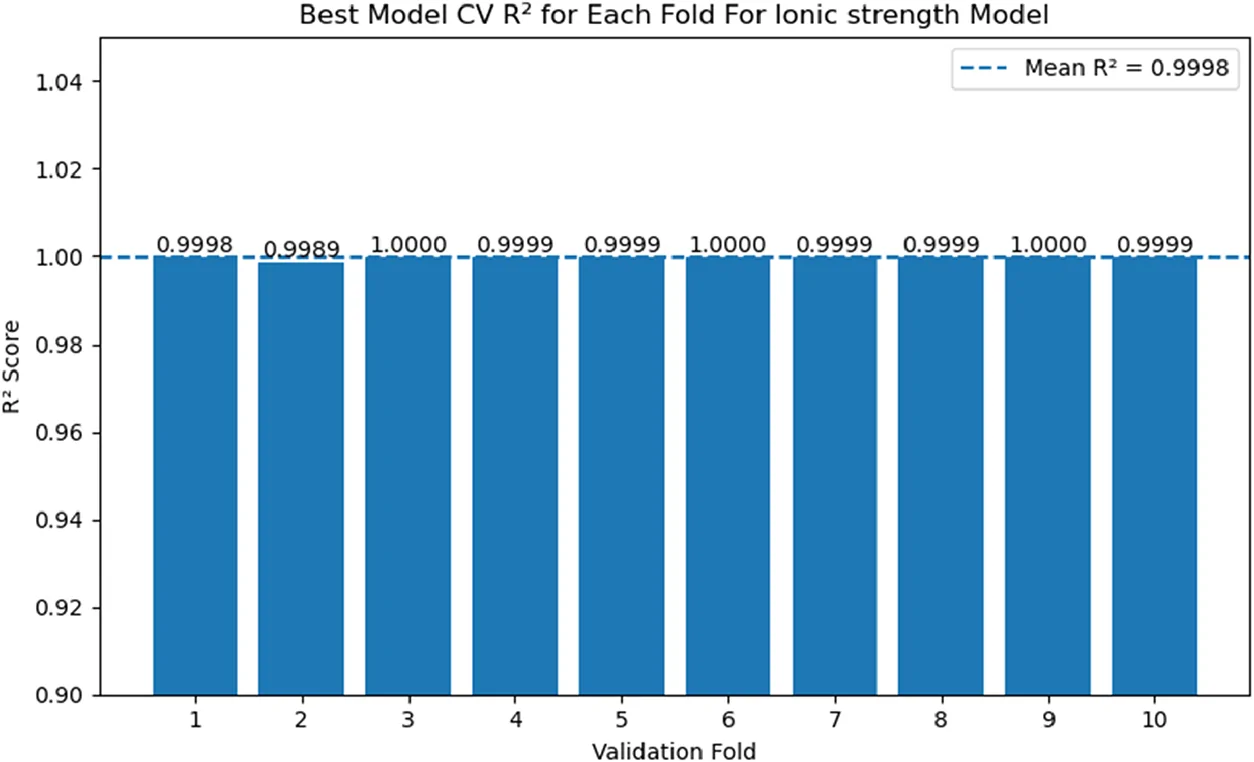
Cross-validation *R* ^2^ scores for the optimized Extra Trees model across all ten folds for the ionic-strength data set.

### 3.7 Overall Comparative Analysis

To finalize the evaluations, the test errors for critical pH and CSC were ranked together by model group using the test set paired RMSE and MAE comparisons shown in Figure 17. This representation gives a more visual comparison of the overall model’s performance on both pH and Csc studies. This evaluation is essential for fines-migration screening, where both pH and ionic strength are dominant factors for the fines destabilization that may occur under different operational scenarios, which require the same level of predictive reliability.

**Figure 17:**
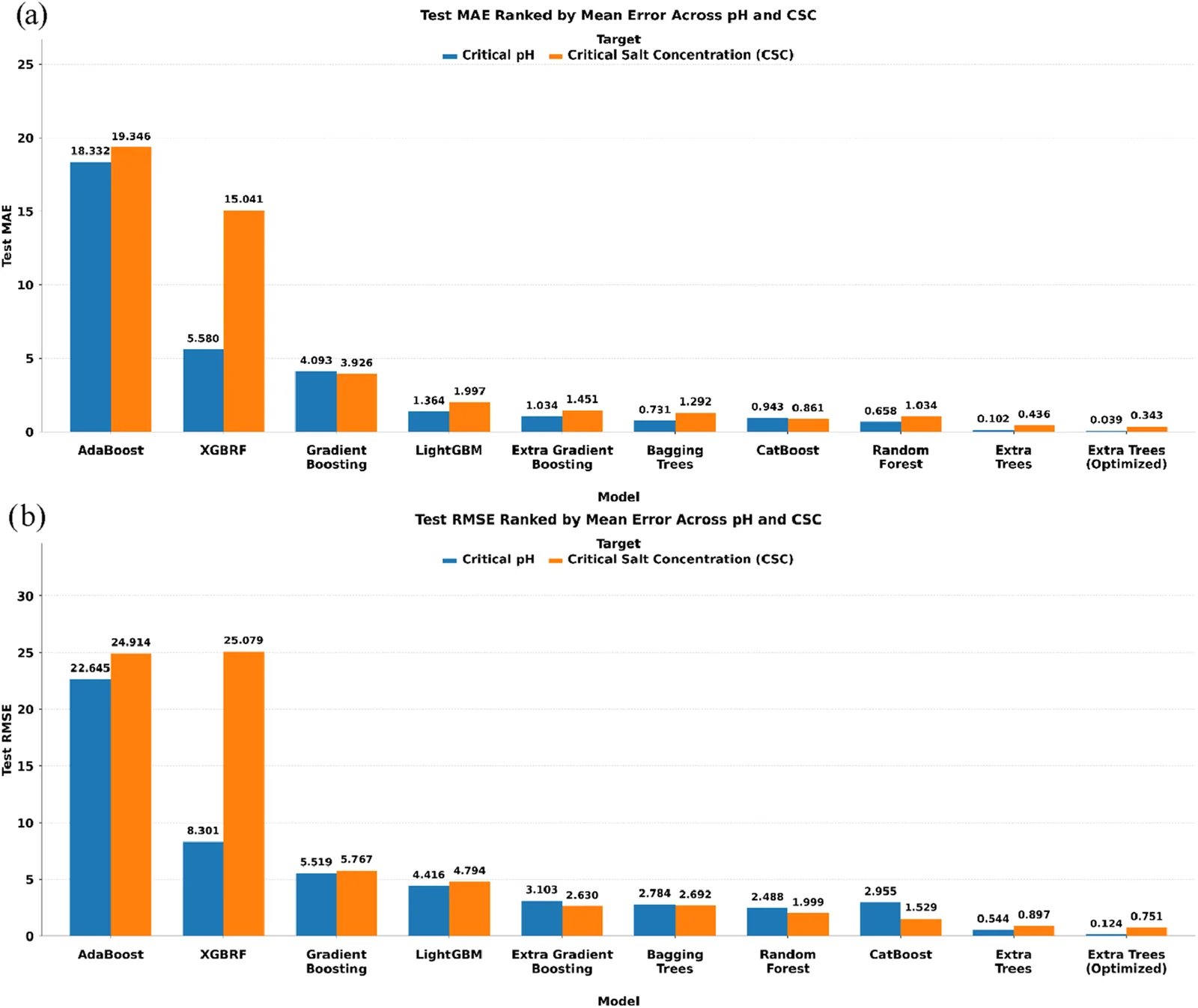
Comparative test-error ranking of ensemble models for critical pH and critical salt concentration prediction using (a) MAE and (b) RSME.

The coupled ranking shows that the best-performing models are not only those with the lowest individual errors but those with the smallest imbalance between pH and CSC predictions. This is important because a model that exhibits a strong performance for one target but weaker performance for the other would not be practically useful. In this comparison, the optimized Extra Trees Ensemble model retains the lowest error across both outputs.

The RMSE and MAE provide different viewpoints on the model’s predictive accuracy. RMSE is more sensitive to outliers, whereas MAE reflects the average magnitude of prediction error. Therefore, models that rank poorly in RMSE relative to MAE produce large errors, which may be problematic near fines-mobilization thresholds (where the total potential is close to zero). Ultimately, models that remain highly ranked under both metrics are of greater practical reliability.

Finally, the optimized Extra Trees model remains the most balanced model across both the parameters, confirming that the optimization improved the overall performance without overfitting on either target.

### 3.8 Practical Field-Screening Workflow

In a field-screening application, the reservoir engineer provides readily available brine descriptors, including pH and ionic strength, together with the known sandstone or mineralogical contexts. In the present model, temperature is treated as a domain-checking variable unless temperature-dependent DLVO or zeta potential data are included during training, in which the optimized model has to be retrained.^68,78^ When zeta potential measurements are unavailable, the already deployed model uses pH and ionic strength within the calibrated sandstone–brine domain represented by the DLVO data set.^56,57^

The trained surrogate predicts V_t_(h) across the prescribed separation-distance grid for candidate pH or salinity values. The threshold module then identifies CSC or critical pH by locating the zero-crossing or selected DLVO stability criterion and reports a threshold uncertainty.^56,58^ The screening output can be classified as stable, transition risk, or unstable.

## 4 Limitations and Domain of Applicability

The proposed framework is a physics-informed surrogate trained on the DLVO-generated data. Its accuracy is therefore bounded by the assumptions, parameter ranges, and calibration quality of the DLVO simulations used to generate the training labels.^57,58^ The high test-set and cross-validation accuracy demonstrate strong interpolation within the sampled DLVO domain, but it does not by itself establish universal field-scale generalization.^68,70^

A key limitation concerns the ζ-potential. In the deployable base model, ζ-potential is excluded because it is highly collinear with pH or ionic strength in the present data sets and is less readily available than routine brine descriptors. This improves field usability but reduces mechanistic completeness.^78,79^ In real reservoirs, zeta potential depends on mineralogical composition, clay type, ion identity, ion valence, specific adsorption, pH, ionic strength, and temperature.^68,78,79^ Consequently, the model is not extrapolated to substantially different mineralogies, multivalent-ion brines, high-salinity adsorption regimes, or temperature-dependent electrokinetic systems without recalibration or additional descriptors.^56,79^

A second limitation is that the data sets are DLVO-simulated that are validated using direct experiments.^56–58^ The surrogate reproduces the DLVO model with high fidelity but does not independently validate the DLVO assumptions under all reservoir conditions.^56,57^

## 5 Conclusions

In summary, the results show that the tree-based ensemble models can reproduce the DLVO-derived total interaction potential with exceptionally high accuracy in both pH- and salinity-controlled regimes, provided that the model complexity and regularization are adequately adjusted. The Extra Trees regressor delivered the best and most stable predictions for this class on independent test sets with superior *R* ^2^, RMSE, and MAE values for both key pH and CSC goals than boosted and bagged ensembles. Bayesian hyperparameter adjustment using Optuna resulted in a further small boost in projected accuracy and cross-validation consistency, indicating that the baseline ensemble designs were already near to their optimal operating point. The predicted-vs-actual plots, residual distributions, and kernel density comparisons suggest that the optimized Extra Trees models are near-perfect with the DLVO targets, homoscedastic, zero-centered residuals, and no visible overfitting, supporting their use as physically consistent surrogates for fines-migration threshold prediction across a wide range of reservoir conditions. These results support the use of the models as rapid DLVO surrogates within the investigated parameter spaces. A key limitation is the use of DLVO-generated rather than direct experimental data. Consequently, the models inherit DLVO assumptions and do not capture the geological heterogeneity, pore-scale transport, hydrodynamic effects, or experimental uncertainty.

The main computational advantage of the surrogate is its fast and cost-efficient screening after training, where the predictions from the deployed Extra Trees models require very low computational costs and can be generated in a very short time. Direct DLVO analysis requires repeated evaluation of van der Waals, electrostatic double-layer, and Born-repulsion terms across salinity or pH and separation-distance grids, often using system-specific electrokinetic inputs,^56,57^ ML surrogate models in reservoir engineering have demonstrated the value of separating one-time training cost from low-cost inference for multiscenario screening and optimization.

## Supplementary Material



## Data Availability

The data sets used and/or analyzed during the current study are available from the corresponding author on reasonable request.

## Notes

Ranges of varied parameters and fixed physicochemical constants used to generate DLVO-based data sets (Table S1) and practical examples of predicting the critical salt concentration and critical pH using experimental data (Figure S1) (PDF)
