# Supplementary material for: Physics-Guided Machine Learning for Predicting Critical Salt Concentration and pH in Sandstone Reservoirs
Source: ACS Omega. 2026 Aug 20;11(34):51731–51. doi: 10.1021/acsomega.6c06045 (PMC13625075; doi:10.1021/acsomega.6c06045)
Supplement: Supplementary file 1 [file ao6c06045_si_001.pdf]

Supporting Information

Physics-Guided Machine Learning for Predicting Critical Salt Concentration and pH in Sandstone Reservoirs

Sami Abderraouf Belkhir[1], *Rizwan Muneer*[2], *Mariam Shakeel*[3], *Younes Alblooshi*[1,*], *Muhammad Rehan Hashmet*[1]

[1] Department of Chemical and Petroleum Engineering, United Arab Emirates University, 15551 Al Ain, United Arab Emirates.

[2] College of Engineering and Science, Hamad Bin Khalifa University, Education City, Doha, Qatar.

[3] Department of Civil and Environmental Engineering, College of Engineering, Qatar University, Doha, Qatar.

[*] Corresponding author: y.alblooshi@uaeu.ac.ae

Table S1. Physicochemical and dataset parameters used to generate the DLVO-derived CSC and critical-pH datasets

Parameter	Symbol / unit	CSC dataset	Critical-pH dataset	Treatment in the ML workflow	Selection basis
Ionic strength	I (mol L^{-1})	0.04–0.20	0.2	Varied predictor for the CSC dataset	Range adopted from the validated salinity-dependent DLVO dataset [8,32].
pH	pH (–)	7	3–9	Varied predictor for the critical-pH dataset	Range adopted from the validated pH-dependent DLVO dataset [11,41].
Zeta potential	ζ (mV)	−35.5 to −29.5	−25.5 to −7.0	Used in DLVO data generation; excluded from the final ML feature set after multicollinearity analysis	Values correspond to the experimentally established salinity- or pH-dependent electrokinetic relationships.
Surface separation distance	h (nm)	0.25–10	0.05–0.75	Varied and retained as an ML predictor	Selected to resolve the short- and intermediate-range changes in the DLVO interaction-energy profile.
Total interaction potential	Vₜ (kT)	Calculated using the DLVO formulation	Calculated using the DLVO formulation	Response variable predicted by the ML models	Defined as the combined London–van der Waals, electrical-double-layer, and Born-repulsion contributions.
Number of generated observations	N	2,850	4,163	Used for model training, cross-validation, and independent testing	Generated over the specified physicochemical grids.
Fine-particle radius or diameter	aₚ (nm)	900	900	Held constant during dataset generation	Required for reproducibility because particle size affects the magnitude of surface interaction energy.
Hamaker constant	A_H (J)	1.49E-20	1.49E-20	Held constant during dataset generation	Controls the magnitude of London–van der Waals attraction.
Temperature	T (K)	297.15	297.15	Held constant during dataset generation	Experimental temperature because it affects thermal energy and electrostatic properties.
Permittivity of free space	ε_o (F. m^{-1})	8.85E-12	8.85E-12	Held constant or calculated from temperature	Required in the electrical-double-layer contribution.
Dielectric constant for water	ε_m (–)	80	80	Held constant during dataset generation	Required in the electrical-double-layer contribution.
Atomic collision diameter	σ (nm)	5E-10	5E-10	Held constant during dataset generation	Required only when the short-range Born-repulsion term is explicitly included.
Boltzmann constant	k_B (J K^{-1})	1.38E-23	1.38E-23	Held constant during dataset generation	Required for London–van der Waals attraction.
Electron charge	e (C)	1.60E-19	1.60E-19	Held constant during dataset generation	Required for Deby length calculation.
Avogadro's Number	N_A ($mole^{-1}$)	6.02E23	6.02E23	Held constant during dataset generation	Required for Deby length calculation.

Note: Yellow cells identify fixed DLVO values that are not reported in the current ML manuscript. Complete these cells directly from the original DLVO calculation files or source studies [8,11,32,41] before submission; the values should not be estimated. Zeta potential was used to generate the physics-labelled datasets but was removed from the final ML feature sets because of strong multicollinearity with ionic strength or pH.

Abbreviations: CSC, critical salt concentration; DLVO, Derjaguin–Landau–Verwey–Overbeek; EDL, electrical double layer; ML, machine learning.

Equations Used in DLVO modeling

The London van Der Waals (V_{LVW}) Attractive Potential

$$V_{LVW} = -\frac{A_H}{6}\left[\frac{2(1+H)}{H(2+H)} + \ln\left(\frac{H}{2+H}\right)\right]$$

Electric Double Layer (V_{EDL}) Repulsive Potential

$$V_{EDL} = 2\pi\varepsilon_m\varepsilon_0 a_p \zeta^2 . ln(1 + e^{\frac{-h}{k^{-1}}})$$

Born Repulsion (V_{BR}) Potential

$$V_{BR} = \frac{A_H}{7560}\left(\frac{\sigma}{a_p}\right)\left[\frac{8+H}{(2+H)^7} + \frac{6-H}{H^7}\right]$$

DLVO Model

$$V_t = V_{LVW} + V_{EDL} + V_{BR} + V_{AB}$$

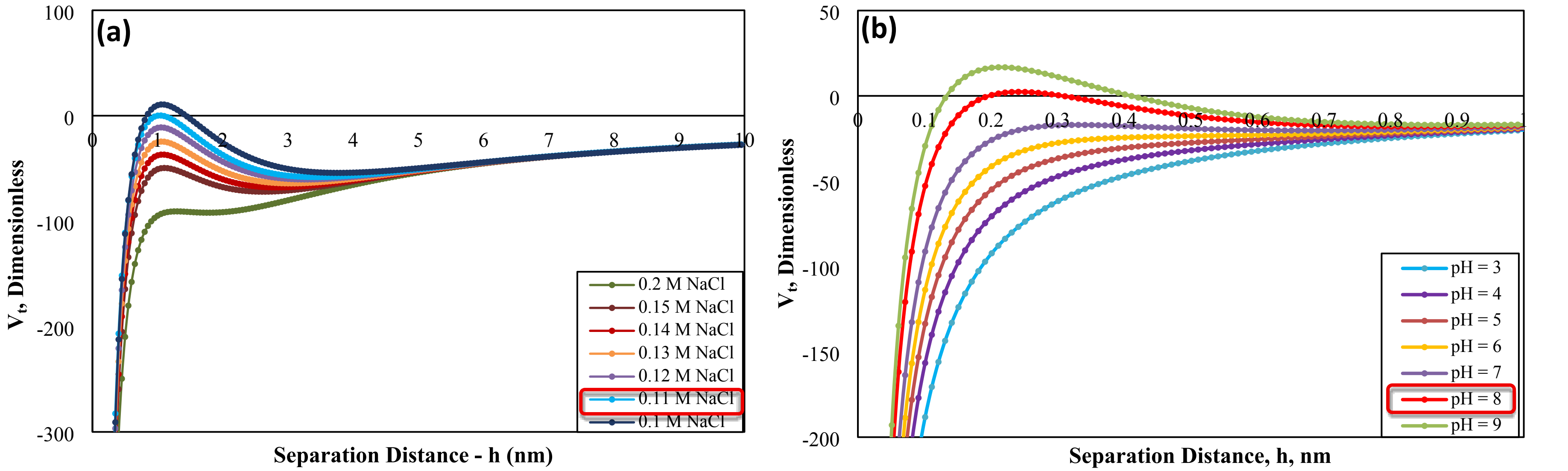

Figure S1. Prediction of the critical salt concentration (CSC) and critical pH using a DLVO model developed with experimental data, (a) prediction of CSC for NaCl, and (b) prediction of critical pH.
